# Expression of pigment epithelium‐derived factor and thrombospondin‐1 regulate proliferation and migration of retinal pigment epithelial cells

**DOI:** 10.14814/phy2.12266

**Published:** 2015-01-19

**Authors:** Mitra Farnoodian, James B. Kinter, Saeed Yadranji Aghdam, Ismail Zaitoun, Christine M. Sorenson, Nader Sheibani

**Affiliations:** Department of Ophthalmology and Visual Sciences, Clinical Investigation Graduate Program, University of Wisconsin, School of Medicine and Public Health, Madison, Wisconsin; Department of Pediatrics, University of Wisconsin, School of Medicine and Public Health, Madison, Wisconsin; McPherson Eye Research Institute, University of Wisconsin, School of Medicine and Public Health, Madison, Wisconsin; Department of Biomedical Engineering, University of Wisconsin, School of Medicine and Public Health, Madison, Wisconsin

**Keywords:** Adhesion, cell proliferation, migration, oxidative stress, RPE cells

## Abstract

Age‐related macular degeneration (AMD) is the leading cause of vision loss among elderly. Although the pathogenesis of AMD is associated with retinal pigmented epithelium (RPE) dysfunction and abnormal neovascularization the detailed mechanisms remain unresolved. RPE is a specialized monolayer of epithelial cells with important functions in ocular homeostasis. Pathological RPE damage contributes to major ocular conditions including retinal degeneration and irreversible loss of vision in AMD. RPE cells also assist in the maintenance of the ocular angiogenic balance by production of positive and negative regulatory factors including vascular endothelial growth factor (VEGF), thrombospondin‐1 (TSP1), and pigment epithelium‐derived factor (PEDF). The altered production of PEDF and TSP1, as endogenous inhibitors of angiogenesis and inflammation, by RPE cells have been linked to pathogenesis of AMD and choroidal and retinal neovascularization. However, lack of simple methods for isolation and culture of mouse RPE cells has resulted in limited knowledge regarding the cell autonomous role of TSP1 and PEDF in RPE cell function. Here, we describe a method for routine isolation and propagation of RPE cells from wild‐type, TSP1, and PEDF‐deficient mice, and have investigated their impact on RPE cell function. We showed that expression of TSP1 and PEDF significantly impacted RPE cell proliferation, migration, adhesion, oxidative state, and phagocytic activity with minimal effect on their basal rate of apoptosis. Together, our results indicated that the expression of PEDF and TSP1 by RPE cells play crucial roles not only in regulation of ocular vascular homeostasis but also have significant impact on their cellular function.

## Abstract (precis)

Here, we report the isolation of RPE cells from wild‐type and transgenic mice retina. We demonstrate that lack of thrompospondin‐1 or pigment epithelium‐derived factor impacts the proliferation, migration, adhesion, oxidative state, and phagocytic activity of these cells.

## Introduction

Age‐related macular degeneration (AMD) is one of the major causes of visual impairment in the elderly population worldwide. Despite the high prevalence of AMD, the etiology of this disease remains largely unknown. AMD is presented in two major forms, the dry form which is associated with degeneration of retinal pigment epithelium (RPE), and the exudative or wet form which presents the formation of choroidal neovascularization (CNV) (Hua et al. 2012).The impairment of RPE cell function is an early and crucial event in the molecular pathways leading to clinically relevant AMD changes associated with increased production of vascular endothelial growth factor (VEGF) and CNV. However, the detailed mechanisms impacting RPE cell function and pathogenesis of AMD remain poorly defined.

The retinal pigment epithelium is a single layer of epithelial cells with essential roles in photoreceptor function and defensive immune mechanism of macula (Korte et al. 1984). The RPE cells are involved in phagocytosis of photoreceptor outer segments, oxidative stress response, photoreceptor renewal, and preservation of their photo transduction (Korte et al. 1984; Sparrow et al. 2010; Klettner 2012). The RPE cells have evolved a preventative mechanism against high levels of antioxidants and photo‐oxidation through light filtration and absorption (Strauss 2005; Kevany and Palczewski 2010). In addition, RPE cells selectively regulate the transport of metabolites, ions, nutrients, and water between the retina and choriocapillaris, and maintain outer retinal‐blood barrier (Strauss 2005). The failure of RPE cell function results in major ocular clinical changes such as retinal degeneration and irreversible vision loss (Korte et al. 1984; Yang et al. 2006a,b).

The RPE cells, as a major source of ocular angioregulatory proteins, play an important role in maintaining the ocular angiogenic homeostasis through a balanced production of positive and negative regulatory factors including VEGF, thrombospondin‐1 (TSP1), and pigment epithelium‐derived factor (PEDF). Altered production of these factors in RPE cells may contribute to various eye diseases with a neovascular component including diabetic retinopathy (DR) and exudative AMD (Campochiaro 2000; Duh et al. 2004; Strauss 2005; Sparrow et al. 2010). The increased production of VEGF has been identified as an essential factor in the development and progression of CNV. In addition, many studies have reported impaired production of TSP1 and PEDF in vascular retinopathies including diabetic retinopathy and exudative AMD. However, how these factors impact RPE cell function needs further investigation.

In human eyes, TSP1 is present at high levels in the vitreous and is a major component of the Bruch's membrane (Sheibani et al. 2000; Uno et al. 2006). Decreased TSP1 expression in Bruch's membrane and choroidal vessels during AMD is suggested as a potential regulatory factor, which favors the formation of CNV (Uno et al. 2006). Expression of TSP1 is crucial for normal postnatal development of retinal vasculature and CNV (Wang et al. 2003, 2009, 2012). TSP1 is a homo‐trimeric matricellular protein produced by various cell types including RPE cells (Adams and Lawler 2004; He et al. 2006). It modulates proliferation, migration, differentiation, and apoptosis in a cell type‐specific manner. Thus, TSP1 plays a critical role in the regulation of various biological functions including vascular homeostasis, immunity, and wound healing (Sheibani and Frazier 1999; Mochizuki et al. 2013; Masli et al. 2014). Appropriate production of TSP1 contributes to maintenance of the optical clarity and normal ocular angiogenesis function of the eye (Hiscott et al. 1996a,b; Sheibani et al. 2000; Saika et al. 2004).We previously reported that pathogenesis and progression of DR due to TSP1 deficiency may be influenced by expression of PEDF (Sorenson et al. 2013). Thus, a relationship between TSP1 and PEDF expression may exist.

A number of studies have demonstrated important roles for PEDF in modulation of vascular leakage and angiogenesis in AMD and DR (Papapetropoulos et al. 1997; Yang et al. 2006a,b; Rizzolo et al. 2011). A significant decrease in the PEDF plasma level has been demonstrated in patients with the dry form of AMD. In contrast in the wet AMD patients, a strong positive correlation between VEGF and PEDF concentrations was observed (Machalińska et al. 2012). PEDF, a glycoprotein (50 kDa), is also an endogenous inhibitor of angiogenesis and is present in the vitreous at high levels (Karakousis et al. 2001). PEDF was originally described by its ability to stimulate differentiation of retinoblastoma cells (Barnstable and Tombran‐Tink 2004). However, recent studies have established important roles for PEDF in inflammation, maintenance of normal extracellular matrix, promotion of cell attachment, stability of the endothelium of choriocapillaris, and neurite outgrowths (Yamagishi et al. 2003; Bernard et al. 2009). It also protects retinal neurons from light damage and oxidative stress, as well as inhibiting EC migration and growth of new blood vessels (Bhutto and Lutty 2012). PEDF is also considered as a crucial factor associated with absence of vascularity in the cornea, vitreous, and outer retina (Barnstable and Tombran‐Tink 2004). We have previously shown that mouse RPE cells are a major source of PEDF production (Al‐Shabrawey et al. 2011). How PEDF impacts RPE cell function and contributes to pathogenesis of AMD and CNV requires further elucidation.

It is now clearly established that RPE cells play a key role in the development and stabilization of retinal structure, and maintenance of the ocular angiogenic balance (Strauss 2005). These activities are accomplished through production of numerous factors but little is known about how these factors impact RPE cell function. These studies are hampered by the lack of availability of methods to readily culture RPE cells from wild‐type and transgenic mice retina. Although, RPE cells have been cultured from mouse retina these cells have limited proliferation capacity and/or require special conditions (Kato et al. 1996; Martin et al. 2009), which limit many biochemical studies that require large number of cells. In the present study, we describe a method for routine culturing of RPE cells from wild‐type, and TSP1 and PEDF‐deficient mice, which can be readily manipulated in culture and maintained for many passages without a significant impact on the expression of their specific markers. Using these cells we show, for the first time, the cell autonomous impact of PEDF and TSP1 on RPE cell function. We found that lack of TSP1 or PEDF in RPE cells impacted their proliferation, adhesion, migration, oxidative state, and phagocytic activity with minimal impact on their basal rate of apoptosis. Collectively, our results indicate important roles for PEDF and TSP1 in modulation of RPE cell functions critical to the role of RPE cells as an important care provider and regulator of various ocular functions. Our results also indicate that these proteins not only play an important role in ocular vascular homeostasis, but also have important role in RPE cell function further emphasizing a key role for these proteins in the pathogenesis of AMD and CNV.

## Material and Methods

### Experimental animals

All the experiments were conducted in accordance with the Association for Research in Vision and Ophthalmology statement for the use of Animals in Ophthalmic and Vision Research and were approved by the Institutional Animal Care and use Committee of the University of Wisconsin School of Medicine and Public health. Immorto mice expressing a temperature‐sensitive simian virus (SV) 40 large T antigen were obtained from Charles Rivers Laboratories (Wilmington, MA). TSP1−/− and PEDF−/− mice on a C57BL/6J background were generated and maintained as previously described, and crossed with immorto mice backcrossed to C57BL/6J mice (Lawler et al. 1998; Su et al. 2003). The isolated DNA from tail biopsies was used for screening of various transgenes as previously described (Su et al. 2003; Scheef et al. 2009).

### Isolation and culture of RPE cells

RPE cells were isolated form one litter (6 or 7 pups) of 4‐week‐old wild‐type, PEDF−/− and TSP1−/− Immorto mice (all on C57BL/6J background) by removing retinas, and optic nerve form the posterior portion of the eyes, cutting the remaining cup and collection of RPE layer under a dissecting microscope. RPE sheets were digested in 5 mL of Collagenase type I (Worthington, Lakewood, NJ; 1 mg/mL in serum‐free Dulbecco ҆ s modified Eagle ҆ s medium (DMEM)) and incubated at 37°C for 20 min. The digested tissues were washed with DMEM containing 10% fetal bovine serum (FBS), and resuspended in 0.5 mL of RPE cell growth medium [DMEM containing 10% FBS, 2 mmol/L L‐glutamine, 100 *μ*g/mL streptomycin, 100 U/mL penicillin and murine recombinant INF‐*γ* (R& D Systems, Minneapolis, MN) at 44 U/mL]. Cells were plated in a well of 24‐well plate coated with fibronectin (2 *μ*g/mL in serum‐free DMEM; BD Bioscience, Bedford, MA) and incubated in a tissue culture incubator at 33°C with 5% CO_2_. Cell were progressively passaged to larger plates and maintained in 60‐mm tissue culture plates coated with 1% gelatin. For all experiments, cells were incubated with RPE cell growth medium containing INF‐*γ* in a tissue culture incubator at 33°C with 5% CO_2_. However, to confirm the observed results is specifically due to PEDF and/ or TSP1 deficiency, cells were also incubated with RPE cell growth medium without INF‐*γ* in a tissue culture incubator at 37°C with 5% CO_2_ for 48 h to eliminate large T antigen. Cells allowed to reach 80–90% confluence and then used for experiments. For some experiments cells were allowed to reach confluence (junctional organization) and used for experiments a week later. Three different isolations of RPE cells were used in these studies and all cells were used prior to passage 20.

### FACS analysis

RPE cells form 60‐mm culture plates were rinsed with PBS containing 0.04% EDTA and incubated with 1.5 mL of Cell Dissociation Solution (Sigma, St. Louis, MO). Cells were then washed, collected from plates with DMEM containing 10% FBS, centrifuged, and blocked in 0.5 mL of Tris‐buffered saline (TBS; 25 mmol/L Tris‐ HCl, 150 mmol/L NaCl, pH 7.6) with 1% goat serum for 20 min on ice. Cells were then pelleted and incubated in 0.5 mL TBS with 1% BSA containing a specific primary antibody on ice for 30 min. The following antibodies were used anti‐RPE65 (MAB 5428), anti‐bestrophin (MAB 5466), anti‐VCAM‐1 (CBL1300), anti‐endoglin (CBL1358), anti‐*β*3 (MAB 1957), anti‐*α*5*β*1 (MAB 1999), anti‐*α*v*β*3 (MAB 1976Z), anti‐*α*2 (AB1936), anti‐*α*3 (AB1920), anti‐*α*5 (AB1921), anti‐*α*V integrins (MAB 1930) (Millopore, Billerica, MA), anti‐ ICAM‐1(SC‐1511), anti‐*β*5 (SC‐5401), anti‐*β*8‐ integrins (SC‐10817) (Santa Cruz Biotechnology, Santa Cruz, CA), anti‐ICAM‐2, anti‐ *α*1‐integrin (BD Biosciences), anti‐VEGF receptor‐1 (VEGFR‐1), anti‐VEGFR‐2 (R&D Systems), and anti‐ CD47, anti‐ PDGF‐R*α*, and anti‐ PDGF‐R*β* (eBioscience, San Diego, CA) antibodies at dilutions recommended by the supplier. Cells were then rinsed twice with TBS containing 1% BSA and incubated with appropriate FITC‐conjugated secondary antibody (Pierce, Rockford, IL) prepared in TBS containing 1% BSA for 30 min on ice. Following incubation, cells were washed twice with TBS containing 1% BSA, resuspended in 0.5 mL of TBS with 1% BSA and analyzed by a FACScan caliber flow cytometer (Becton Dickinson, Franklin Lakes, NJ). These experiments were repeated twice using two different isolations of RPE cells with similar results. The mean fluorescent intensities are indicated for each antibody.

### Cell proliferation studies

Cell proliferation was assessed by counting the number of cells for two weeks. Cells (1 × 10^4^) were plated in multiple sets of gelatin‐coated 60‐mm tissue culture plates, fed every other day for the duration of experiment. The number of cells was determined by counting every other day, on days not fed, in triplicates. The rate of DNA synthesis was also assessed using Click‐It EDU Alexa Flour 488 as recommended by the supplier (Life technologies, Grand Island, NY). The assay measures DNA synthesis using 5‐ethynyl‐2′‐deoxyuridine (EdU) a nucleoside analog of thymidine. The percentage of cells undergoing active DNA synthesis was determined by FACScan caliber flow cytometry (Becton Dickinson).

TdT‐dUPT Terminal Nick‐End Labeling (TUNEL) was used to assess apoptotic cell death. TUNEL staining was performed using Click‐iT‐TUNEL Alexa Flour imaging assay as recommended by supplier (Life Technologies). A similar experiment was performed in the presence of 50 *μ*mol/L H_2_O_2_ (Fisher Scientific). This concentration was determined based on moderate effect on cell viability after 24–48 h. Positive apoptotic cells counted in 10 high‐power fields (×200) and calculated as percentage of total cell number. All samples were prepared in triplicates and repeated twice.

### Indirect immunofluorescence studies

Cells (1 × 10^5^) were plated on fibronectin‐coated 4‐well chamber slides (5 *μ*g/mL in PBS) and allowed to reach confluence (1–2 days). Cells were rinsed with PBS, fixed with cold acetone for 10 min on ice, permeabilized with PBS containing 0.1% Triton X‐100 for 12 min at room temperature, and then blocked with PBS containing 1% BSA at 37°C for 30 min. Following incubation, slides were washed once with PBS and incubated with specific primary antibodies for 2 h at room temperature. The primary antibodies were anti ‐ZO‐1 (Life Technologies), anti‐ *β*‐catenin, anti‐P120‐ catenin, anti‐N‐cadherin (BD Bioscience), anti‐vinculin and FITC‐conjugated phalloidin (Sigma) prepared in PBS (1:200) containing 1% BSA. Following incubation, slides were washed with PBS and incubated with specific Cy3‐conjugated secondary antibodies (Jackson ImmunoResearch, West Grove, PA; (1:800) in PBS containing 1% BSA for 1 h at room temperature. Following incubation, slides were washed four times with PBS and examined using a fluorescence microscope (Carl Zeiss Optical, Germany) and images were taken in digital format.

### Scratch wound assays

Cells (1 × 10^6^) were plated in 60‐mm tissue culture dishes and allowed to reach confluent (1–2 days). Plates were wounded using a 1‐mL micropipette tip, washed with growth medium twice to remove detached cells, and fed with growth medium containing 1 *μ*mol/L 5‐fluorouracil (Sigma) to block cell proliferation. Wound closure was monitored by phase microscopy at different time points (0, 24, 48 h) and images were captured in digital format. The migrated distance as percentage of total distance was determined for quantitative assessment of data as described previouslyDiMaio and Sheibani (2008).

### Transwell migration assays

Cell migration was also determined using a transwell migration assay. Costar transwell inserts (8‐*μ*m pore size, 6.5‐mm membrane, Lowell, MA) were coated with PBS containing fibronectin (2 *μ*g/mL) on the bottom side at 4°C overnight. After washing with PBS, inserts were blocked in PBS containing 1% BSA for 1 h at room temperature and rinsed with PBS. Cells were trypsinized and resuspended in serum‐free medium, and 1 × 10^5^ cells/0.1 mL was added to the top of inserts. The inserts were placed in a 24‐well plate containing 0.5 mL of serum‐free medium and incubated for 4 h at 33°C. Following incubation, cells were fixed with 2% paraformaldehyde for 10 min at room temperature stained with hematoxylin and eosin (H& E), and the inserts were mounted on a slide with the cell facing down. The number of cells migrated through the membrane was determined by counting 10 high‐power fields (×200). Each experiment was done in triplicates and repeated with two different isolation of RPE cells.

### PEDF and TSP1 reexpression studies

To express PEDF in PEDF−/− RPE cells, 2.5 × 10^5^ cells were plated in 35‐mm tissue culture dishes. The next day, lenti viruses encoding PEDF or GFP (100 pfu/cell, 20 *μ*L) and 20 *μ*L of lenti booster (Sirion Biotech, Martinsried, Germany) including 10 *μ*L each of 1:100 dilution of solution A and B in PBS, were mixed and diluted in 500 *μ*L of opti‐MEM (Life Technologies) and incubated for 15 min at room temperature. Following incubation, the tissue culture plates were washed twice with serum‐free medium and incubated with 0.5 mL of lenti virus and lenti booster mixture overnight. The next day medium containing lenti virus and lenti booster mixture were removed, fresh growth medium was added to the plates and incubated for 4 days before they were used for further analysis.

To express TSP1 in TSP1−/− RPE cells, 2.5 × 10^5^ cells were plated in 35‐mm tissue culture dishes. The next day, adenoviruses encoding TSP1 or GFP (625 pfu/cell; 50 *μ*L) and adeno booster (10 *μ*L, Sirion Biotech) were mixed and diluted in 500 *μ*L of opti‐MEM (Life Technologies) and incubated for 15 min at room temperature. Following incubation, the tissue culture plates were washed twice with serum‐free medium and incubated with 0.5 mL of adenovirus and adeno booster mixture overnight. The next day, medium containing virus and booster mixture were removed, fresh growth medium was added to the plates and incubated for 3 days before they were used for further analysis.

### Cell adhesion assays

Cell adhesion assays were conducted using 96‐well plates (Maxisorb Nunc Immunoplate, Fisher Scientific) coated with different concentration of collagen I, collagen IV, vitronectin and fibronectin (BD Biosciences), diluted in TBS (50 *μ*L/well) containing 2 mmol/L CaCl_2_, 2 mmol/L MgCl_2_ (Ca/Mg) overnight at 4°C. Plates were rinsed four times with TBS containing Ca/Mg (200 *μ*L/well), blocked using TBS with Ca/Mg containing 1% BSA (200 *μ*L/well) at room temperature for 1 h. Cells were collected from tissue culture plates using 2 mL of dissociation buffer (2 mmol/L EDTA, 0.05% BSA in TBS), rinsed with TBS and resuspended in cell‐binding buffer (150 mmol/L NaCl, 20 mmol/L HEPES, 4 mg/mL BSA, pH 7.4) at ~5 × 10^5^ cells/mL. The coated plates were washed with TBS containing Ca/Mg, and incubated with equal amount (50 *μ*L/well) of cell suspension and TBS with Ca/Mg for 2 h at 37°C. Following incubation, plates were washed with TBS with Ca/Mg to remove nonadherent cells. The number of adherent cells was quantified by measuring intracellular acid phosphatase activity as described previouslyPark et al. (2010). These assays were performed in triplicates and repeated with two different isolations of RPE cells.

### Western blot analysis

Cells (1 × 10^6^) were plated in 60‐mm tissue culture dishes until reached confluence (~90%). Cells were washed with serum‐free DMEM medium and incubated in growth medium without serum (conditioned medium, CM) for 2 days. The CM was centrifuged at 400 × g for 5 min to remove cell debris and stored at −80°C for further analysis. Cell lysates were also prepared using 100 *μ*L of lysis buffer (50 mmol/L HEPES pH 7.5, 1 mmol/L MgCl_2_, 1 mmol/L CaCl_2_, 100 mmol/L NaCl, and 0.1 mmol/L EDTA with 1% NP‐40, 1% Triton X‐100, and protease inhibitor cocktail; Roche Biochemicals, Mannheim, Germany). BCA protein assay (Bio‐ Rad, Hercules, and CA) was used to determine protein concentration. Samples were mixed with appropriate amount of 6× SDS buffer and analyzed by 4–20% SDS‐PAGE (Invitrogen). Proteins were transferred to nitrocellulose membrane and blocked in TBS containing 0.05% Tween 20 (TBST) with 5% skim milk for 1 h at room temperature. Membranes were incubated with primary antibody for 2 h at room temperature, washed with TBST, and incubated with appropriate horseradish peroxidase‐conjugated secondary antibody (1:10,000; Jackson ImmunoResearch) for 1 h at room temperature. The following antibodies were used: anti‐ fibronectin (SC‐9068), anti‐eNos (SC‐654), anti‐ FAK (SC‐558), anti‐c‐Src (SC‐8056) (Santa Cruz, Biotechnology), anti‐TSP1 (A 6. 1, Neo Markers, Fermont, CA), anti‐TSP2, anti‐ PEDF, anti‐cathepsin B (R& D System), anti‐tenascin C (AB19013), anti‐ Collagen IV (AB756P) (Millipore), anti‐SPARC, anti‐opticin, anti‐ periostin, and anti‐ MFGE‐8 (R& D System), anti‐iNOS, anti‐PDI, anti‐p‐Src, anti‐p‐P38, anti‐P38, anti‐p‐AKT1, anti‐AKT1, anti‐p‐ERK and anti‐ ERK (Cell Signaling), anti‐nNOS (BD Bioscience), and anti‐*β*‐ actin (Thermo Fisher) were used at dilutions recommended by the supplier. The protein bands were visualized using enhanced chemiluminescence reagent (GE Bioscience, Piscataway, NJ). The mean band intensities were determined using Image J 1.46a (National Institute of Health, Bethesda, MD) and normalized against β‐actin band as loading control.

### Phagocytosis assays

Phagocytosis activity was assayed using the unique pHrodo^™^ – based system (Life technologies) based on the acidification of the particles as they are ingested. pHrodo^™^ dye is a fluorogenic dye that greatly increases in fluorescence intensity when the pH becomes more acidic. RPE cells in 35‐mm culture plates were loaded with medium containing pHrodo^™^ Green *E. coli* BioParticles conjugates and incubated for different time points (5 and 24 h). Following incubation, cells were rinsed with PBS containing 0.04% EDTA, and incubated with 1.5 mL of cell dissociation solution (Sigma). Cells were then washed, collected from plates, washed twice with PBS, resuspended in 0.5 mL of PBS, and analyzed by a FACScan caliber flow cytometry (Becton Dickinson).

### Proteasome peptidase assays

Proteasome peptidase assays were performed as previously described by us Aghdam et al. (2013). The cells were collected and lysed using the lysis buffer containing 50 mmol/L Tris‐HCl pH 7.4, 1 mmol/L ATP, 10% glycerol, 0.1% NP40, 2 mmol/L MgCl_2_, 1.5 mmol/L DTT, and 0.03% SDS. BCA protein assay (Bio‐Rad) was used to determine protein concentrations. The protein lysates (50 *μ*g/well) were added into a dark 96‐well microplate and probed with the fluorogenic peptide substrates: Z‐Leu‐Leu‐Glu‐AMC for caspase‐like, Ac‐Arg‐Leu‐Arg‐ AMC for trypsin‐like, and Suc‐Leu‐Leu‐Val‐Tyr‐AMC for chymotrypsin‐like (Enzo Life Sciences, Farmingdale, NY) at the final concentration of 100 *μ*mol/L in a total volume of 0.1 mL. The plates were incubated in the dark for 30 min at 37°C. Following incubation, the reaction was stopped using 100% ethanol (100 *μ*L/well). The fluorescent intensity was measured using a plate reader (PerkinElmer, Wellesley, MA). The excitation and emission wavelengths were 355 and 460 nm, respectively.

### Secreted VEGF levels

The level of VEGF produced by WT, TSP1−/−, and PEDF−/− RPE cells were determined using the Mouse VEGF Immunoassay Kit (R& D Systems). Cells (1 × 10^6^) were plated in 60‐mm tissue culture dishes and allowed to reach 90% confluence. The cells were washed with serum‐free DMEM and incubated with 2 mL of growth medium without serum for 2 days. The CM were collected, centrifuged at 400 × g for 5 min to remove cell debris, and used for VEGF measurements as recommended by the supplier.

### Capillary morphogenesis assays

Tissue culture plates (35 mm) were coated with 0.5 mL Matrigel (10 mg/mL, BD Biosciences) and incubated at 37°C for at least 30 min to allow the gel harden. Mouse choroidal EC (ChEC) were prepared and maintained as previously described by us Lavine et al. (2013). The ChEC were removed by trypsin‐EDTA, washed with DMEM containing 10% FBS. Cells (1 × 10^5^ cells/mL) were resuspended in collected conditioned medium (CM) form wild type, PEDF−/− and TSP1−/− RPE cells. Cells (2 mL) were applied to the Matrigel‐coated plates, incubated at 37°C, photographed after 14 h using a Nikon microscope in a digital format. For quantitative assessment of the data, the mean number of branch points was determined by counting the branch points in five high‐power fields (×100).

### RNA purification and real‐time qPCR analysis

The total RNA from RPE cells was extracted using mirVana PARIS Kit (Invitrogen). cDNA synthesis was performed from 1 *μ*g of total RNA using Sprint RT Complete‐Double PrePrimed Kit (Clontech, Mountain View, CA). One microliter of each cDNA (dilution 1:10) was used as template in qPCR assays, performed in triplicate of three biological replicates on Mastercycler Realplex (Eppendorf, Hauppauge, NY) using the SYBR‐Green qPCR Premix (Clontech). Amplification parameters were as follows: 95°C for 2 min; 40 cycles of amplification (95°C for 15 sec, 60°C for 40 sec); dissociation curve step (95°C for 15 sec, 60°C for 15 sec, 95°C for 15 sec). Primer sequences for different inflammatory cytokines were: TNF‐*α* 5′‐ACCGTCAGCCGATTTGCTAT‐3′ (forward) and TNF‐*α* 5′‐TTGACGGCAGAGAGGAGGTT‐ 3′ (reverse), IL‐18 5`‐AAGAAAATGGA GACCTGGAATCAG‐3` (forward) and IL‐18 5′‐ATTCCGTATTACTGCGGTTGTACA‐3′ (reverse), MCP‐1 5′‐GTCT GTGCTGACCCCAAGAAG‐3′ (forward) and MCP‐1 5′‐TGGTTCC GATCCAGGTTTTTA‐3′ (reverse), and RANTES 5′‐GCCCACGTCAAGGA GTATTTCT‐3′ (forward) and RANTES 5′‐CAAACACGA CTGCAAGATTGGA‐3′ (reverse).

Standard curves were generated from known quantities for each of the target gene of linearized plasmid DNA. Ten times dilution series were used for each known target, which were amplified using SYBR‐Green qPCR. The linear regression line for ng of DNA was determined from relative fluorescent units (RFU) at a threshold fluorescence value (Ct) to quantify gene targets from cell extracts by comparing the RFU at the Ct to the standard curve, normalized by the simultaneous amplification of RpL13a, a housekeeping gene. The following primers for RpL13A 5`‐TCTCAAGGTTGTTCGGCTGAA‐3′ (forward) and RpL13A 5′‐CCAGACG CCCCAGGTA‐3′ (reverse) were used.

### Ex vivo sprouting of RPE‐choroid complex

Choroidal explants were prepared and cultured as described previously Kobayashi et al. (1998), with some modifications. Briefly, postnatal day 21 (P21) mice were anesthetized using isoflurane and killed by cervical dislocation. Eyes were enucleated, washed three times, and kept in ice‐cold serum‐free DMEM. Attached tissues to the outer surface of the eyeball (blood vessels, fatty and connective tissues) were shaved in ice‐cold DMEM under a dissection microscope. The cornea, lens, and corpus vitreum were removed before the intermediate segment containing the sclera, choroid, RPE, and the retina was dissected along the whole circumference. The neuroretinal was removed before the sclera, and the RPE‐choroid complex were sectioned into 0.5–1.0 mm pieces. These pieces were finally transferred into 35‐mm culture dishes coated with 0.5 mL of Matrigel (10 mg/mL) (BD Biosciences) and allowed to harden. Tissue preparations were transferred into a 37°C cell culture incubator without medium for 20 min. Endothelial cell growth medium (DMEM containing 10% FBS, 2 mmol/L L‐glutamine, 2 mmol/L sodium pyruvate, 20 mmol/L HEPES, 1% nonessential amino acids, 100 *μ*g streptomycin, 100 U/mL penicillin, 55 U/mL heparin, and endothelial growth supplement 100 *μ*g/mL; Sigma) was then added (2 mL/dish) and incubated at 37°C with 5% CO_2_ for eight days. Explants were fed every other day. After 8 days, preparations were fixed in 4% PFA for 30 min at room temperature, washed three times in PBS before they were imaged using a Nikon microscope. Ten explants per eye were prepared and cultured in a single dish, and at least three mice per genotype were used.

### NO measurements

The intracellular NO levels produced by WT, TSP1−/−, and PEDF −/− RPE cells was determined using 4‐amino‐5‐methylamino‐2, 7‐difluoroflourescein diacetate (DAF‐FM diacetate; Invitrogen). DAF‐FM is produced after deacetylation of DAF‐FM diacetate by intracellular esterases. The increased fluorescence intensity of DAF‐FM due to the reaction with NO is measured with a fluorescein filter. Cells (5 × 10^3^ cells/0.1 mL) were plated on gelatin‐coated 96‐well black/clear bottom plates (BD Biosciences) and incubated overnight. The next day, medium was removed and 0.1 mL of RPE medium containing 30 *μ*mol/L DAF‐FM was added to each well and incubated for 40 min. The medium was replaced with fresh RPE medium and incubated for an additional 30 min. Following incubation, the cells were washed with TBS (0.1 mL, twice) and fluorescence intensity was measured at an excitation of 458 nm and an emission of 535 nm using a fluorescent microplate reader (Victa21420 Multilabel Counter, PerkinElmer). This assay was conducted in triplicates and repeated with two different isolation of RPE cells.

### Wholemount staining studies

RPE layers were prepared form 4‐week‐old wild‐type, PEDF−/−, and TSP1 −/− mice. Eyes were fixed in 4% PFA in PBS for 2 h. Following fixation, RPE, choroid, and sclera complex was prepared by removing the retina and cutting the optic nerve from the posterior portion of the eyes. The remaining cup was washed with PBS three times for 10 min, and blocked in blocking solution (50% FCS, 20% normal goat serum; NGS, in PBS containing 0.01% Triton 100X) at RT for 1 h. After blocking samples were washed with PBS three times for 10 min and incubated with primary antibodies including anti‐iNOS, anti‐HNE (Cell Signaling), anti‐eNOS (SC‐654) (Santa Cruz), and anti‐ZO‐1 (Life Technology) in PBS containing 20% FCS, 20% NGS, and 0.01% TX‐100 overnight at 4°C. Samples were then washed with PBS and incubated with specific Cy3‐conjugated secondary antibody (1:800; Jackson ImmunoResearch) in PBS containing 20% FCS, 20% NGS, and 0.01% TX‐100 at room temperature for 2–4 h. Following incubation, samples were washed five times with PBS, flattened by four radial cuts, mounted on the slide, and examined using a fluorescence microscope (Carl Zeiss Optical) and the images were taken in digital format.

### Assessments of reactive oxygen species

The level of cellular reactive oxygen species was measured with dihydroethidium staining (DHE). DHE is a weak blue fluorescent dye which binds to DNA upon oxidation to red fluorescent ethidium by 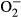. Cells (3 × 10^4^) were plated on fibronectin‐coated chamber slide (5 *μ*g/mL; BD Bioscience) and incubated for 24 h in growth medium. Following incubation, the cells were exposed to 10 *μ*mol/L DHE for 15–20 min, washed with growth medium and incubated twice with growth medium each for 30 min. A similar experiment was performed in the presence of 50 *μ*mol/L H_2_O_2_ (positive control; Fisher Scientific). Digital images were captured at the same exposure time for all samples. For quantitative analysis, images were analyzed with Image J software (National Institute of Health, Bethesda, MD). The mean fluorescent intensity was determined from intensities of multiple cells (100 cells). A similar experiment was performed using cells treated with 50 *μ*mol/L H_2_O_2_ for 24 h, and three independent experiments with two different isolation of RPE cells were performed.

### Statistical analysis

Statistical differences between control and treated samples were evaluated using Graphpad Prism software (La Jolla, CA) according to the Tukey Multiple Comparison test with a *P* value <0.05 considered significant.

## Results

### Isolation and characterization of PEDF−/− and TSP1−/− RPE cells

**Figure 1.**
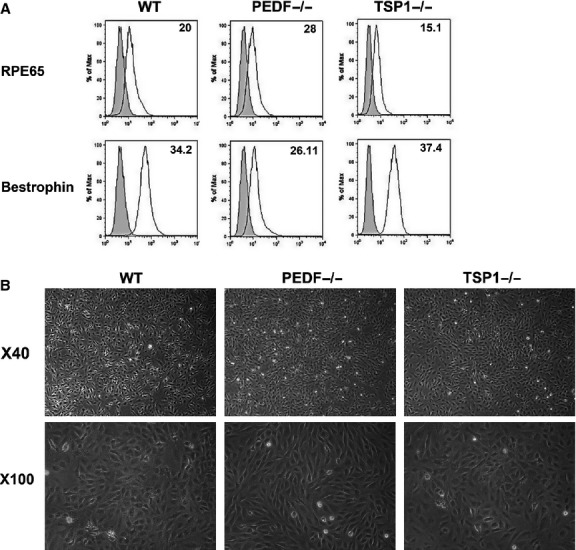
Isolation and characterization of mouse retinal pigment epithelium (RPE) cells. Wild‐type (WT) RPE, PEDF−/−, and TSP1−/− RPE cells were prepared as described in Material and Methods and plated in 60‐mm tissue culture dishes coated with 1% gelatin. (A) Expression of specific RPE cells markers. RPE cells were examined for expression of RPE65 and Bestrophin by FACS analysis. The shaded area shows control IgG staining. (B) Phase micrographs of RPE cells. Images were captured in digital format at ×40 and ×100 magnifications. These experiments were repeated using three different isolations of RPE cells with similar results.

RPE cells were prepared from 4‐week‐old wild‐type, TSP1−/− and PEDF−/− Immorto mice as described in Material and Methods. The expression of RPE‐specific markers was confirmed by FACS analysis (Fig. 1A). All cells expressed significant amount of bestrophin and RPE 65, as expected. The results are representative of sample mean from three different isolations, and there were no statistically significant differences between the cells. The PEDF−/− RPE cells exhibited a more spindle‐shaped and elongated morphology compared to wild‐type cells on gelatin‐coated plates, while TSP1−/− RPE cells exhibited a similar morphology as wild‐type RPE cells (Fig. 1B).

**Figure 2.**
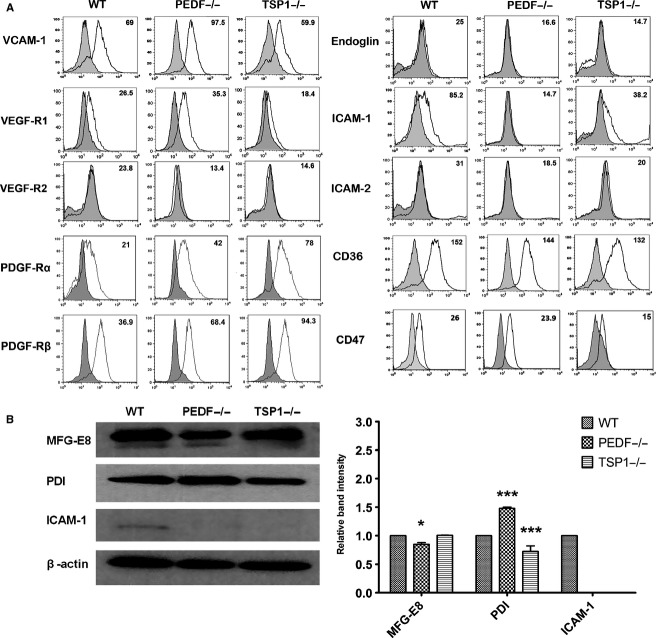
Expression of other RPE cell markers. (A) RPE cells were examined for expression of VCAM‐1, VEGF‐R1, VEGF‐R2, Endoglin, ICAM‐1, ICAM‐2, CD36, PDGF‐R*α*, PDGF‐R*β*, and CD47by FACS analysis. The shaded area shows control IgG staining. (B) Cell lysates were prepared from wild‐type (WT), PEDF−/−, and TSP1−/− RPE cells and analyzed for expression level of MFG‐E8, PDI, ICAM‐1 and *β*‐actin as loading control by Western blot analysis using specific antibodies. The quantitative assessment of data is shown on the right (*P < 0.05, ***P < 0.001, *n* = 3).

To determine the properties of PEDF−/−, TSP1−/−, and wild‐type RPE cells, we next analyzed the expression of other RPE cell markers by FACS (Fig. 2A) and Western blot (Fig. 2B) analysis. Alteration in some of these markers including (ICAM‐1, VCAM‐1, and VEGF receptors) has been reported as underlying factors in the development of AMD. The decreased expression level of ICAM‐1 was observed in PEDF −/− RPE and TSP1−/− compared with wild‐type cells. However, the PEDF−/− RPE cells represented the most dramatic change compared to wild‐type cells. The expression of other markers including CD36, CD47, VEGFR‐1, and VCAM‐1 was observed in all RPE cells with modest variations among the cell types. We also determined VEGFR‐2, endoglin, and ICAM‐2 expression, which were undetectable in all cells (Fig. 2A). PDGF‐R*β* signaling plays a key role in the RPE cell migration and proliferation through activation of the downstream signaling pathways (Schlingemann 2004). The TSP1−/− RPE cells exhibited a dramatic increase in PDGF‐R*β* expression compared with PEDF−/− and wild‐type cells (Fig. 2A). Similarly increased levels of PDGF‐R*α* expression was observed in TSP1−/− and PEDF−/− RPE cells compared to wild‐type cells.

In the retina, the secreted milk fat globule‐EGF factor 8 (MFG‐E8) is involved in clearance of shed photoreceptor outer segment (POS) by ligating *α*v*β*5 integrin. Absence of either MFG‐E8 ligand or *α*v*β*5 receptor results in abolished RPE phagocytosis (Finnemann et al. 1997; Nandrot et al. 2007). MFG‐E8 also plays an important role in apoptotic cell clearance, angiogenesis, and adaptive immunity (Deroide et al. 2013). MFG‐E8 protein levels were examined by Western blot analysis. We observed a modest decrease in expression level of MFG‐E8 in PEDF−/−, but not TSP1−/−, RPE cells compared with wild‐type cells. We also examined protein disulfide isomerase (PDI) expression in RPE cells. PDI is a crucial factor for cell viability, which plays a critical function during protein folding (Hahm et al. 2013). The downregulation of PDI in human RPE cells may lead to protein aggregation and endoplasmic reticulum stress (Yokoyama et al. 2006). PEDF−/− RPE cells expressed increased level of PDI compared with wild‐type and TSP1−/− RPE cells. A significant decrease in the level of ICAM‐1 was observed in TSP1−/− and PEDF−/− cells consistent with FACS results.

**Figure 3.**
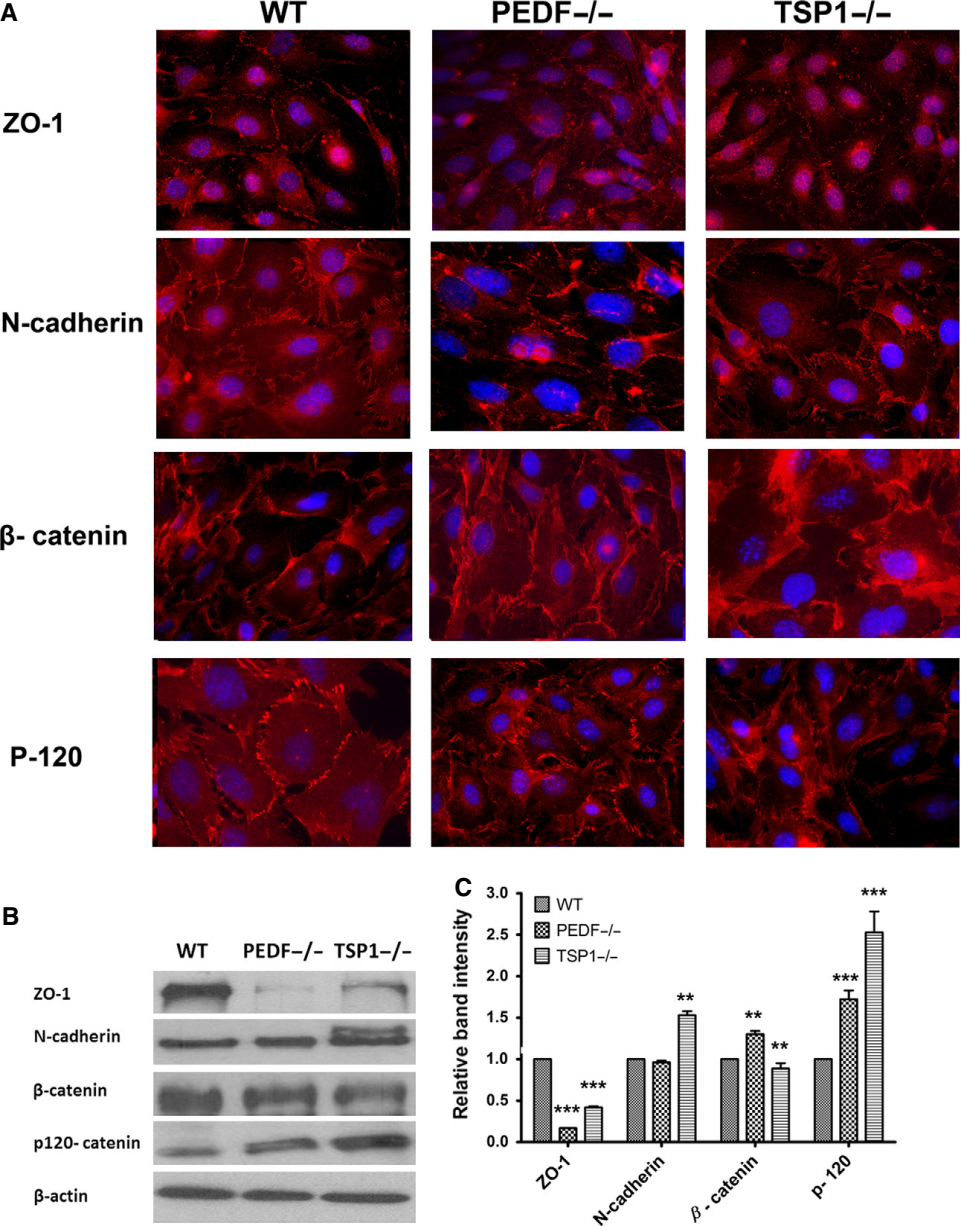
Cellular localization and expression of junctional proteins. (A) The localization of ZO‐1, N‐cadherin, *β*‐catenin, and P‐120 catenin was determined by immunofluorescence staining. The WT, PEDF−/−, and TSP1−/− RPE cells were plated on fibronectin‐coated chamber slides and stained with specific antibodies as detailed in Material and Methods. No staining was observed in the absence of primary antibody (not shown). Please note the absence of N‐cadherin from the sites of cell–cell contact in PEDF−/− RPE cells, as well as reduced junctional localization in TSP1−/− RPE cells. (B) Western blot analysis of junctional proteins. Total cell lysates were prepared from WT, PEDF−/−, and TSP1−/− RPE cells and analyzed for expression level of ZO‐1, N‐cadherin, *β*‐ catenin, P‐120 catenin, and *β*‐actin. Please note decreased expression of ZO‐1 proteins in PEDF−/− RPE cells. The TSP1−/− RPE cells expressed increased levels of N‐cadherin and P‐120 catenin. The *β*‐catenin level was increased in TSP1−/− RPE cells. The *β*‐actin was used for loading control. The quantification of data is shown in (C) (**P < 0.01, ***P < 0.001, *n* = 3). These experiments were repeated using three different isolations of RPE cells with similar results.

Adherens junctions facilitate intercellular adhesions, a process with major roles in maintaining tissue integrity and normal morphology of RPE cells (Bailey et al. 2004).The altered morphology of PEDF−/− RPE cells suggested altered cell–cell interactions. We next determined the localization and expression of junctional protein complexes by indirect immunofluorescence staining (Fig. 3A) and Western blot analysis (Fig. 3B). N‐cadherin is reported as the dominant cadherin in RPE cells with important roles in cell migration and photoreceptor survival, and localization at cell–cell junctions (Kaida et al. 2000; Chen and Ma 2007). Here, we observed lack of N‐cadherin junctional localization in PEDF−/− RPE cells compared with the wild‐type cells (Fig. 3A), while no significant difference was observed in the N‐cadherin expression level compared with wild‐type cells (Fig. 3B and C). TSP1−/− RPE cells showed increased levels of N‐cadherin compared with wild‐type or PEDF−/− cells. In addition, a band with higher molecular weight was detected in TSP1−/− RPE cells for N‐cadherin. Thus, the absence of PEDF and TSP1 was associated with defects in cell–cell interactions and junction formation in RPE cells.

Tight junctions are complex, dynamic structures that regulate cell proliferation, polarity, and paracellular diffusion and permeability. Formation of tight junctions is important for appropriate barrier function of the RPE cells. ZO‐1 plays a major role in the formation and stabilization of tight junctions (Rizzolo et al. 2011). The PEDF −/− and TSP1−/− RPE cells exhibited decreased levels of ZO‐1 (Fig. 3B). However, no significant changes in junctional localization of ZO‐1 were observed in TSP1−/− and PEDF−/− RPE cells compared with the wild‐type cells (Fig. 3A). In addition, ZO‐1 showed enhanced nuclear localization in TSP1−/− RPE cells compared to wild‐type and PEDF−/− cells, without impacting the localization of other components of adherens junction complexes. Similar results were observed in RPE‐choroid complex prepared from TSP1−/− mice in vivo (not shown). We did not observe any effect on ZO‐1 nuclear localization in PEDF−/− RPE cells. Junctional localization of p120‐ catenin, another adherens junctional protein, was similarly observed in all RPE cells. However, an increase in p120‐ catenin level was observed in PEDF−/− and TSP1−/− RPE cells (Fig. 3B). The *β*‐catenin level was significantly increased in PEDF−/− RPE cells compared to wild‐type cells. However, *β*‐catenin localization was not affected by PEDF and TSP1 deficiency in RPE cells (Fig. 3B and C). To roll out the possible impact of large T antigen and INF‐*γ* present in growth medium, similar experiments were conducted in the absence of INF‐*γ* at 37°C to eliminate large T antigen expression with similar results (not shown). Collectively, our results indicated that deficiency in PEDF or TSP1 had a significant impact on junctional properties of RPE cells.

### PEDF−/− and TSP1−/− RPE cells displayed a greater rate of proliferation

**Figure 4.**
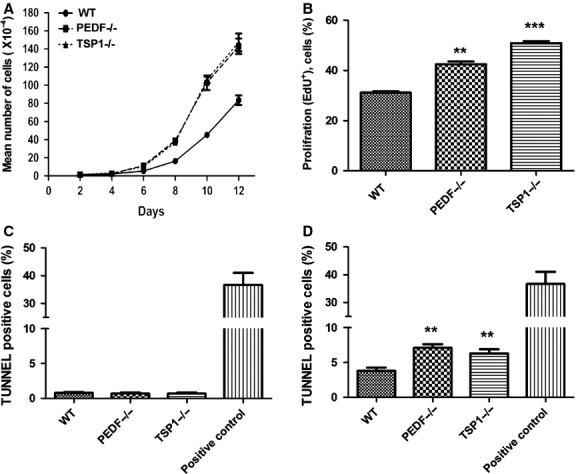
Alterations in proliferation and apoptosis of RPE cells. (A) The rate of proliferation was determined by counting the number of cells. A significant increase in the proliferation of PEDF−/− and TSP1−/− RPE cells was observed compared with WT cells. (B) An increase in the rate of DNA synthesis was observed in TSP1−/− and PEDF−/− RPE cells compared with the WT cells. (C) The rate of apoptosis was determined using TdT‐ dUTP Terminal Nick‐ End Labeling (TUNNEL). A similar experiment was carried out using H_2_O_2_ (50 *μ*Μ), as an inducer of apoptosis (positive control). Please note no differences were observed in the basal rate of apoptosis in these cells. (D) A significant increase in the rate of apoptosis was observed in PEDF−/− and TSP1−/− RPE cells when challenged with H_2_O_2_ (50 *μ*Μ) compared with WT cells (0.05, **P < 0.01, ***P < 0.001, *n* = 3).

RPE cell integrity is essential for proper retina function. The demonstrated impact of PEDF and TSP1 on junctional properties of RPE cells suggested alterations in proliferation of these cells. RPE cells are normally postmitotic in adults and their activation is associated with proliferative vitro retinopathy (PVR) (Hecquet et al. 2002). Choroidal neovascularization (CNV), a major pathologic condition in patient with AMD, at the early stage is associated with hyperplastic RPE cells. However, in the late stages of CNV the RPE cells proliferate in a monolayer to enclose CNV and promote CNV regression (Bhutto and Lutty 2012).The rate of cell proliferation was determined by counting the number of cells for two weeks. An increase in the rate of proliferation was observed in PEDF−/− and TSP1−/− RPE cells compared to the wild‐type cells (Fig. 4A). We next examined whether the increase in rate of proliferation was the result of enhanced DNA synthesis. The percentage of cells undergoing active DNA synthesis was determined by EdU labeling, a synthetic nucleoside analog. The rate of DNA synthesized in PEDF−/− and TSP1−/− RPE cells was significantly greater than that observed in wild‐type cells (Fig. 4B). Thus, PEDF or TSP1 deficiency is associated with enhanced RPE cell proliferation.

The apoptosis of RPE cells plays an important role in the development of different retinal degenerative diseases (Mascarelli et al. 2001). Human AMD studies suggest that RPE cells, photoreceptors, and inner nuclear layer cells die by apoptosis (geographic atrophy; GA) (Dunaief et al. 2002). We next determined whether lack of PEDF and TSP1 in RPE cells affects their rate of apoptosis. TUNEL staining was used to determine the rate of apoptosis in RPE cells under basal and challenged conditions. A similar rate of apoptosis was observed in all RPE cells at the basal level (Fig. 4C). However, under stress conditions (50 *μ*mol/L H_2_O_2_) PEDF−/− and TSP1−/− RPE cells exhibited significantly higher rate of apoptosis compared with wild‐type cells (Fig. 4D; *P* < 0.05; *n* = 5). Thus, lack of TSP1 or PEDF results in increased sensitivity of RPE cells to oxidative challenge.

### PEDF−/− and TSP1−/− RPE cells were less migratory

**Figure 5.**
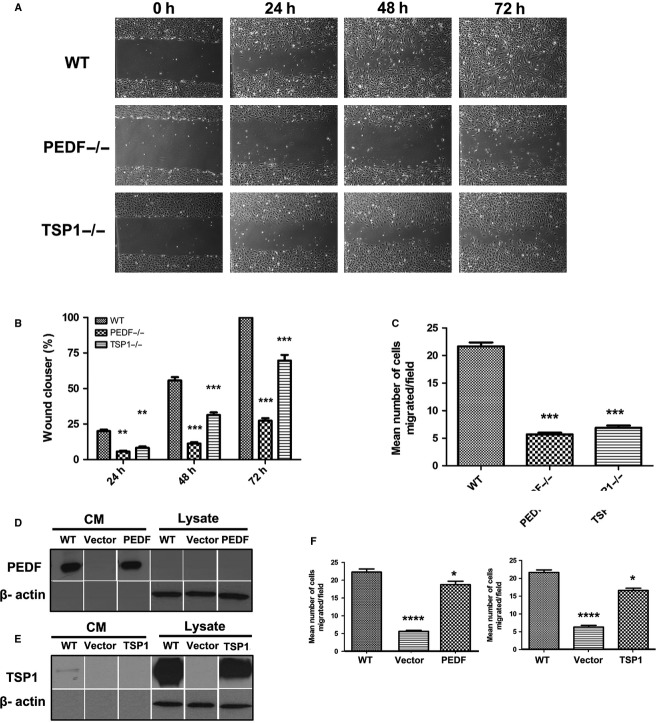
Attenuation of migration in PEDF−/− and TSP1−/− RPE cells. (A) Scratch wound assay of RPE cell monolayers on gelatin‐coated plates was used to determine cell migration. Wound closure was observed by phase microscopy at indicated time points. (B) The quantitative assessment of wound migration (**P < 0.01, ***P < 0.001, *n* = 3). (C) Migration of RPE cells in transwell assays. Please note a significant decrease in the migration of PEDF−/− and TSP1−/− RPE cells compared with WT cells (***P < 0.001, *n* = 3). (D and E) The re‐expression of PEDF and TSP1 in PEDF−/− and TSP1−/− RPE cells. PEDF−/− and TSP1−/− cells were infected with viruses encoding PEDF or TSP1 cDNAs as detailed in Material and Methods. The expression of PEDF and TSP1 was confirmed by Western blot analysis. These lanes are all from the same membrane and extraneous lanes were removed for uniform labeling of the Figure. (F) Reexpression of PEDF and TSP1 in PEDF−/− and TSP1−/− RPE cells restored their migration to near WT RPE cell as determined by transwell migration assay (*P < 0.05, ****P < 0.0001, *n* = 3).

Migration and proliferation of RPE cells contribute to a number of pathologies including PVR (Pastor et al. 2002). Scratch wound assays were conducted to investigate the impacts of PEDF or TSP1 deficiency on RPE cell migration. A significant delay in wound closure was observed in PEDF−/− and TSP1−/− RPE cells compared to wild‐type cells (Fig. 5A). The quantitative assessment of the data is shown in Fig. 5B. Similar results were observed using a transwell migration assay (Fig. 5C).

### Reexpression of PEDF or TSP1 restored normal migration of PEDF−/− and TSP1−/− RPE cells

PEDF−/− and TSP1−/− cells displayed decreased migration compared with wild‐type cells. To examine if reexpression of PEDF or TSP1 is sufficient to restore normal migration in PEDF−/− or TSP1−/− cells, we reexpressed PEDF and TSP1 in PEDF−/− and TSP1−/− cells. Reexpression of PEDF and TSP1 was confirmed by Western blot analysis of cell lysates and conditioned medium (Fig. 5D and E). A significant improvement in migration of RPE cells was observed in PEDF−/− and TSP1−/− cells upon reexpression of PEDF and TSP1 (Fig. 5F).

**Figure 6.**
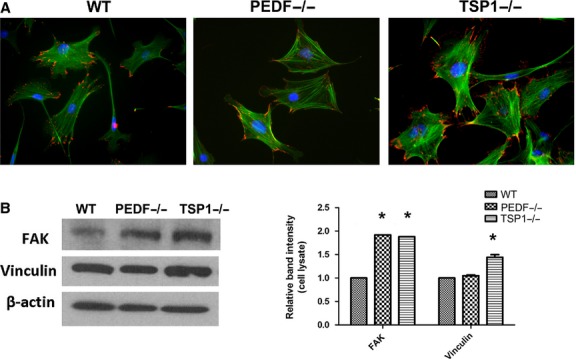
Organization of actin stress fibers and focal adhesions in RPE cells. (A) RPE cells plated on fibronectin‐coated chamber slides were stained with phalloidin (green; actin) and vinculin (red; focal adhesions). Please note peripheral actin staining and basal organization and fewer numbers of focal adhesions in PEDF−/− and TSP1−/− RPE cells compared with WT cells. This is consistent with reduced migratory phenotype of null cells. (B) Increased protein level of focal adhesion kinase (FAK) and vinculin in TSP1−/− RPE cells. The PEDF−/− RPE cells also expressed increased level of FAK. The *β*‐actin was used as a loading control (*P < 0.05, *n* = 3). These experiments were repeated with three different isolations of RPE cells with similar results.

To confirm the change in migratory phenotype of PEDF−/− and TSP1−/− RPE cells, we examined the formation of actin stress fibers and focal adhesions using indirect immunofluorescence staining. Increased number of focal adhesions was observed in PEDF−/− and TSP1−/− RPE cells compared to wild‐type cells (Fig. 6A). The TSP1−/− and PEDF−/− RPE cells were also spread more with stronger peripheral actin staining. These results were further confirmed by increased expression of FAK in PEDF−/− and TSP1−/− RPE cells compared with the wild‐type cells (Fig. 6B). A significant increase in vinculin level was also observed in TSP1−/− RPE cells. These results are consistent with increased presence of focal adhesions, spreading, and reduced migration of TSP1−/− and PEDF−/− RPE cells.

### PEDF−/− and TSP1−/− RPE cells were more adherent

**Figure 7.**
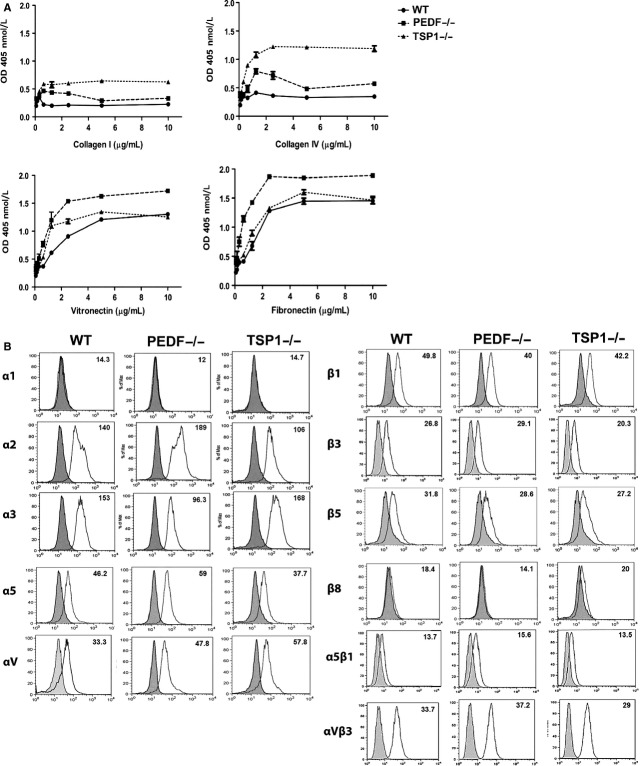
(A) Altered adhesion and expression of integrins in RPE cells. (A) Adhesion of RPE cells to collagen IV, collagen I, vitronectin, and fibronectin was determined as described in Material and Methods. PEDF−/− and TSP1−/− RPE cells adhered stronger to various extracellular matrix (ECM) proteins compared with WT cells. Please note strong adhesion of PEDF−/− RPE cells to vitronectin and fibronectin, and TSP1−/− RPE cells to collagen IV and collagen I. (B) Expression of integrins in RPE cells. *α*1, *α*2, *α*3, *α*5, *α*v, *β*1, *β*2, *β*3, *β*5, *β*8, *α*5*β*1, and *α*V*β*3 integrin expression was determined by FACS analysis as described in Material and Methods. Please note lack of *α*1 expression in RPE cells; while *β*8 integrin was absent in PEDF−/− RPE cells. These experiments were repeated using three different isolations of RPE cells with similar results.

Altered migration in PEDF−/− and TSP1−/− RPE cells suggested changes in their adhesion properties. We next examined the adhesion of RPE cells to various ECM proteins. PEDF−/− RPE cells exhibited stronger adhesion on collagen IV, collagen I, fibronectin, and vitronectin compared with wild‐type cells (Fig. 7A). TSP1−/− RPE cells were also more adherent on fibronectin, vitronectin, collagen IV, and collagen I compared with the wild‐type cells (Fig. 7A).

To determine if changes in adhesion were mediated by alterations in integrin expression, we next determined the expression level of different integrins by FACS analysis. Similar expression levels for *α*2, *α*3, *α*5, *α*v, *β*1, *β*3, *β*5, *α*5*β*1, and *α*v*β*3 were observed in these cells (Fig. 7B). There were no dramatic changes in the integrin expression. However, the possibility of alteration in affinity and avidity of these integrins cannot be excluded. RPE cells had undetectable levels of *α*1 integrin. The PEDF−/− RPE cells showed no detectable expression of *β*8 integrin. However, TSP1−/− and wild‐type RPE cells expressed low levels of *β*8 integrin.

**Figure 8.**
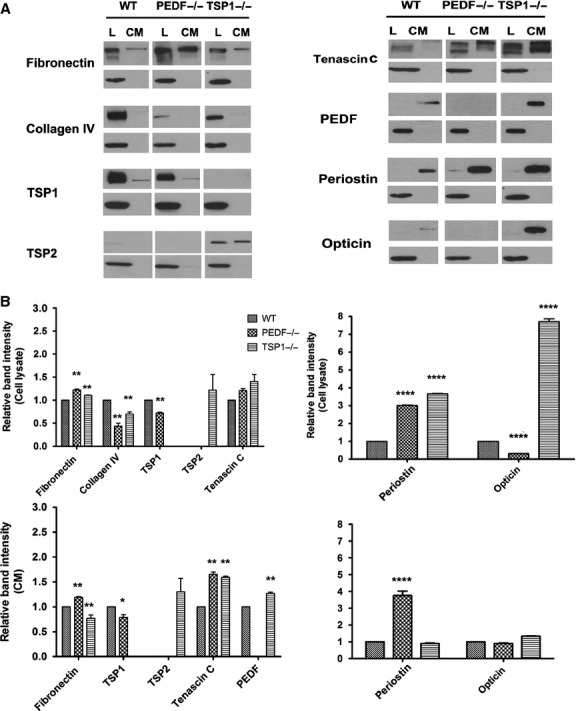
Altered expression of ECM proteins in RPE cells. (A) WT, PEDF−/−, and TSP1−/− RPE cells were plated on gelatin‐coated 60‐mm dishes and incubated for 48 h in serum‐free growth medium. The conditioned medium and cell lysates were collected for analysis of ECM proteins by Western as described in Material and Methods. The expression of fibronectin, collagen IV, tenascin C, TSP1, TSP2, PEDF, periostin, and opticin were determined using specific antibodies. The *β*‐actin was used as a loading control for cell lysates (lower panel). The quantitative assessment of the data is shown in (B) (*P < 0.05, **P < 0.01, ****P < 0.0001; *n* = 3). These experiments were repeated with two different isolations RPE cells with similar results. L: Cell lysate; CM: Conditioned medium.

Diverse biological events including wound closure, inflammatory responses, cell migration, and various developmental processes and angiogenesis are affected by changes in production of various ECM proteins. We next examined the level of various ECM proteins including fibronectin, collagen IV, tenascin C, SPARC, TSP1, TSP2, periostin, opticin, and PEDF in RPE cells (Fig. 8). TSP1−/− and PEDF−/− RPE cells expressed increased levels of fibronectin compared with wild‐type cells. The level of cell associated collagen IV was decreased in PEDF−/− and TSP1−/− RPE cells compared to wild‐type cells. PEDF−/− RPE cells showed a reduction in TSP1 production compared with wild‐type cells. TSP2 expression was only detected in TSP1−/− RPE cells. The PEDF−/− RPE cells showed lower TSP1 production compared with wild‐type cells. In contrast, increased expression of PEDF was observed in TSP1−/− RPE cells compared with wild‐type cells. The TSP1−/− and PEDF−/− RPE cells expressed increased levels of tenascin C in both lysates and CM compared with wild‐type cells. SPARC expression was not affected in RPE cells (not shown). PEDF−/− and TSP1−/− RPE cells exhibited increased periostin expression compared with wild type cells. Opticin was not detectable in PEDF−/− RPE cells while TSP1−/− cells exhibited a dramatic increase in opticin expression compared to wild‐type cells. Thus, our results suggest PEDF and TSP1 expression have major effects on ECM expression impacting adhesive and migratory properties of RPE cells.

### RPE cell phagocytosis

**Figure 9.**
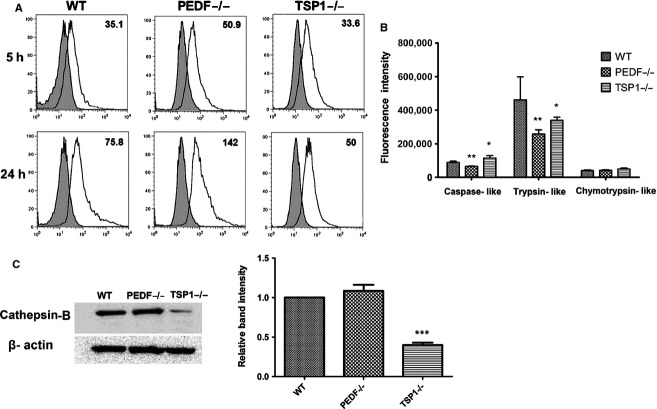
Altered Phagocytic activity in PEDF−/− RPE cells. (A) RPE cells from 35‐mm culture plates were loaded with DMEM medium containing pHrodo TM Green *E. coli* BioParticles conjugates and incubated for different time points (5 and 24 h). Please note the increased phagocytosis activity in PEDF−/− RPE cells compared to WT and TSP1−/− cells. (B) The in vitro proteasome peptidase activity of cultured RPE cells. Chymotrypsin‐like activity was not affected by PEDF and TSP1 deficiency. However, trypsin‐like activity was significantly decreased in PEDF−/− and TSP1−/− RPE cells (*P < 0.05, **P < 0.01, *n* = 3). Please also note a significant decrease in caspase‐like activity in PEDF−/− RPE cells, while TSP1−/− RPE cells exhibited increased caspase‐like activity (*P < 0.05, **P < 0.01, *n* = 3). (C) Attenuation of cathepsin‐B expression in TSP1−/− RPE cells. Please note the dramatic decrease in the level of cathepsin‐B in TSP1−/− RPE cells compared with WT and PEDF−/− RPE cells. These experiments were repeated using three different isolations of RPE cells with similar results.

The phagocytic function of RPE cells is crucial for removal of toxic metabolites and RPE cell survival. Defects in photoreceptor phagocytosis can results in severe retinal pathologies (Sparrow et al. 2010; Mustafi et al. 2013).To determine the impact of PEDF and TSP1 deficiency on RPE cell phagocytic activity, phagocytosis was assayed using the unique pHrodo TM‐based system as detailed in Material and Methods. Enhanced accumulation of phagocytosis activity was observed in PEDF−/− RPE cells compared with wild‐type and TSP1−/− cells (Fig. 9A). A similar trend was observed at later incubation time. Enhanced accumulation of the phagocytized particles may be associated with altered proteasome and/or lysosomal activity (Suraweera et al. 2012).

We next assessed the proteasome activity of RPE cells using an in vitro peptidase assay as detailed in Material and Methods. A decrease in caspase‐like and trypsin‐like activity was observed in PEDF−/− RPE cells compared with wild‐type cells, while TSP1−/− cells exhibited an increase in caspase‐like and trypsin‐like activity compared to the wild‐type cells. All cells similarly showed lower chymotrypsin‐like activity (Fig. 9B). Thus, the lack of PEDF, but not TSP1, in RPE cells was associated with impaired proteasome activity. To determine potential changes in lysosomal system, we also examined the level of cathepsin‐B, the major lysosomal cysteine protease, in lysates prepared from RPE cells by Western blot analysis. PEDF−/− cells expressed similar level of cathepsin‐B as observed in wild‐type cells. In contrast, a dramatic decrease in the level of cathepsin‐B was observed in TSP1−/− RPE cells suggesting reduced lysosomal activity in TSP1−/− RPE cells.

### Alterations in the expression of VEGF and NOS isoforms in RPE cells

**Figure 10.**
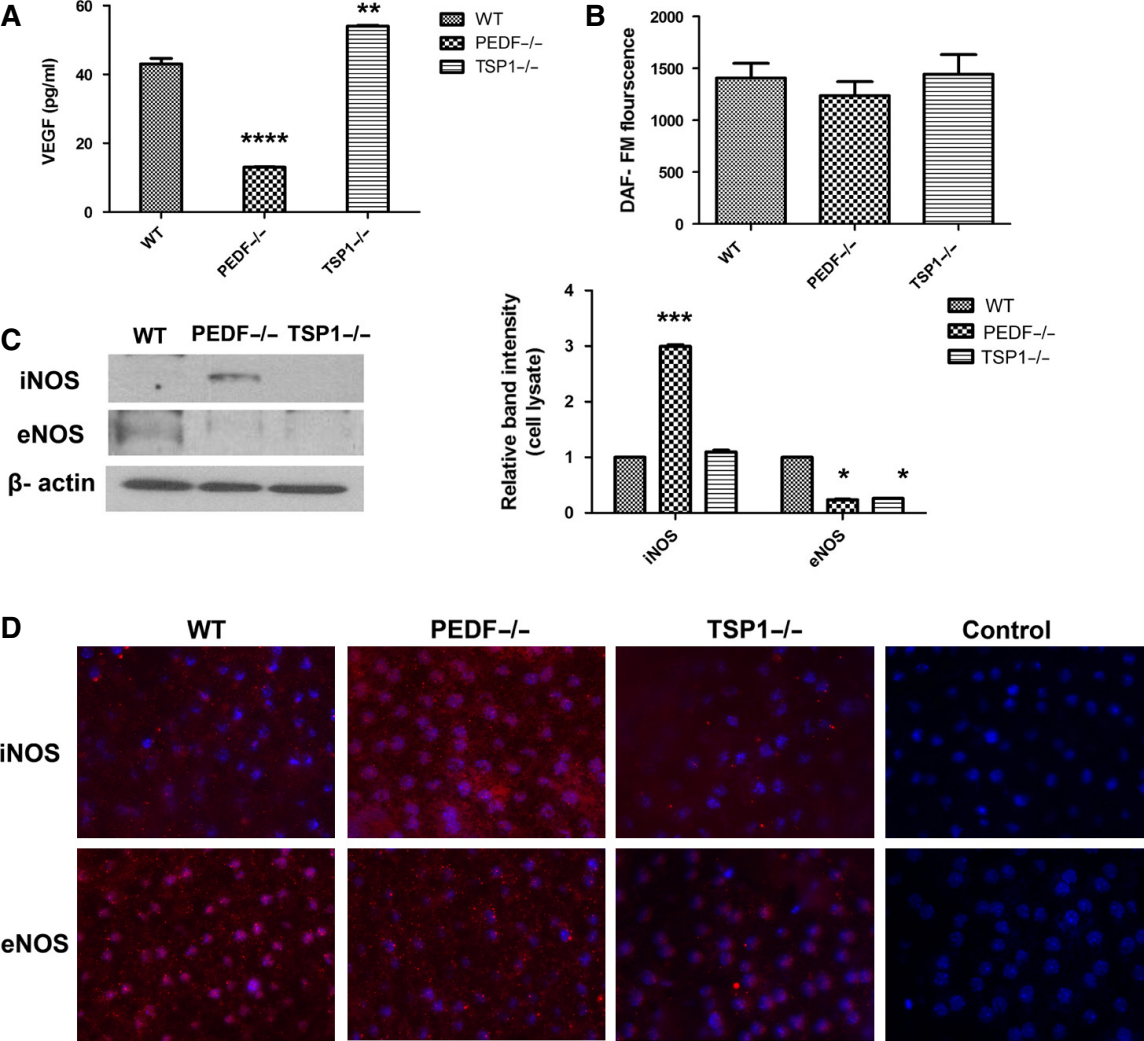
Alterations in VEGF and NOS expression. (A) The level of secreted VEGF was determined in conditioned medium collected from WT, PEDF−/−, and TSP1−/− RPE cells using an ELISA as detailed in Material and Methods. Please note a significant decrease in the level of VEGF produced by PEDF−/− RPE cells compared with WT cells. TSP1−/− RPE cells expressed higher levels of VEGF (**P < 0.01, ****P < 0.0001, *n* = 3). (B) The level of intracellular NO was determined using DAF‐FM as detailed in Material and Methods. Please note similar NO levels in all cells (*P* > 0.05). (C) The levels of iNOS and eNOS were determined by Western blot analysis of cell lysates. Please note a significant increase in the level of iNOS in PEDF−/− RPE cells compared with WT and TSP1−/− RPE cells. Only low level of eNOS was detected in WT RPE cells. The *β*‐actin was used as a loading control (*P < 0.05, ***P < 0.001, *n* = 3). (D) Wholemount staining of RPE layer in RPE‐choroid tissue prepared from PEDF−/−, TSP1−/− and WT mice. Please note increased iNOS staining in RPE‐choroid tissue from PEDF−/− mice compared with TSP1−/− and WT mice. The eNOS was only detectable in WT and to lesser extent in TSP1−/− mice. These experiments were repeated with eyes from five mice with similar results. Control is staining with no primary antibody.

Alterations in the secretory activity of RPE cells are associated with proliferative and degenerative diseases in the retina (Strauss 2005). The increased production of VEGF has been identified as essential factor in the development and progression of AMD and CNV (Kliffen et al. 1997; Yamagishi et al. 2003). Many studies have reported that RPE cells are the major source of VEGF (Adamis et al. 1993; Kliffen et al. 1997; Nagineni et al. 2003). VEGF is considered a survival factor for many cell types including RPE cells (Byeon et al. 2010). To determine the impact of PEDF and TSP1 deficiency on VEGF production, we measured VEGF levels in conditioned medium prepared form PEDF−/− and TSP1−/− RPE cells. A significant decrease in VEGF level was observed in PEDF−/− RPE cells compared to wild‐type cells. In contrast, TSP1−/− RPE cells showed increased VEGF levels compared with wild‐type cells (Fig. 10A). Both PEDF and TSP1 levels are dramatically downregulated in human's exudative AMD samples. Thus, PEDF and TSP1 deficiency have a significant impact on VEGF production in RPE cells and pathogenesis of exudative AMD.

Altered expression of NOS isoforms including reduction in nNOS in RPE nuclei has been reported in AMD (Bhutto et al. 2010). We next examined the expression of various NOS isoforms in PEDF −/− and TSP1−/− RPE cells by Western blot analysis. NO modulates the angiogenic response of various key factors including VEGF (Papapetropoulos et al. 1997). Additionally, NO production is essential for VEGF‐mediated angiogenesis (McLaren et al. 2006). We observed no significant difference in NO levels produced by RPE cells (Fig. 10B).

We detected expression of the inducible NOS (iNOS) in PEDF−/− RPE cells. Wild‐type RPE cells exhibited low level of endothelial NOS (eNOS) expression. While, eNOS expression was undetectable in PEDF−/− and TSP1−/− RPE cells (Fig. 10C). Wholemount staining of RPE/choroid complex from wild‐type, PEDF−/− and TSP1−/− mice showed similar results (Fig. 10D). The expression of nNOS was not detectable in RPE cells by Western blot analysis.

### Capillary morphogenesis of choroidal endothelial cells

**Figure 11.**
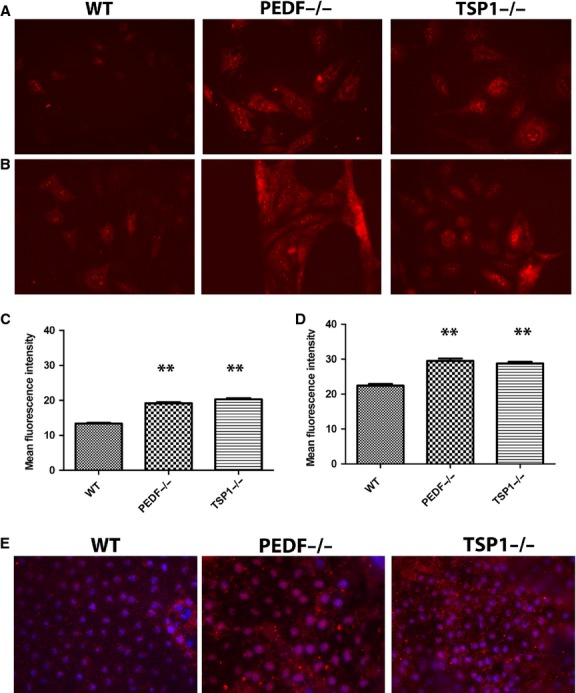
Increased oxidative stress in PEDF−/− and TSP1−/− RPE cells. The level of oxidative stress in RPE cells was assessed by dihydroethidium (DHE) staining under basal (A) or challenged conditions (50 *μ*mol/L H_2_O_2_) (B). A significant increase in the level of ROS was detected under basal (C) and challenged (D) conditions in PEDF−/− and TSP1−/− RPE cells compared to WT cells (**P < 0.01, *n* = 3). (E) Wholemount staining of RPE‐choroid tissue prepared from PEDF−/−, TSP1−/− and WT mice with antibody to 4‐hydroxy‐2‐nanonal (HNE). Please note increased HNE staining in RPE‐choroid tissues from PEDF−/− and TSP1−/− mice compared to Wild type mice. These experiments were repeated using three different isolations of RPE cells, and eyes from five different mice with similar results.

The progression of visual loss in the exudative AMD is associated with abnormal CNV membranes that develop under the retina. This results in leaking serous fluid and blood, and ultimately a blinding disciform scar in and under the retina (Ciulla et al. 1999). We showed that PEDF and TSP1 deficiency differentially impact VEGF secretion in RPE cells. We next determined the effects of conditioned medium collected from PEDF−/− and TSP1−/− RPE cells on capillary morphogenesis of choroidal endothelial cells (ChEC) and ex vivo sprouting of choroid‐RPE complex. Endothelial cells undergo capillary morphogenesis forming a capillary‐like network when plated on Matrigel. This mimics the late stages of angiogenesis (Solowiej et al. 2003). ChEC were isolated and cultured as previously described Lavine et al. (2013). Here, we plated the ChEC, resuspended in CM prepared from wild‐type and, PEDF or TSP1 null RPE cells, on Matrigel. Capillary morphogenesis of ChEC was analyzed and quantified as described in Material and Methods. Conditioned medium from PEDF−/− RPE cells decreased capillary morphogenesis of ChEC compared with wild‐type cells, while CM from TSP1−/− RPE cells increased capillary morphogenesis of ChEC (Fig. 11A and C). These results are consistent with increased production of VEGF by TSP1−/− RPE cells (Fig. 10A). Representative images of capillary morphogenesis are shown in Fig. 11A. We also assessed the ex vivo sprouting of the choroid‐RPE complex prepared from wild‐type, TSP1−/−, and PEDF−/− mice eyes. In contrast to enhanced capillary morphogenesis of ChEC treated with TSP1−/− CM, we observed no significant differences in degree of sprouting angiogenesis in TSP1−/− and PEDF−/− mice compared to wild‐type mice (Fig. 11B and D).

### PEDF and TSP1 deficiency increased ROS generation in RPE cells

**Figure 12.**
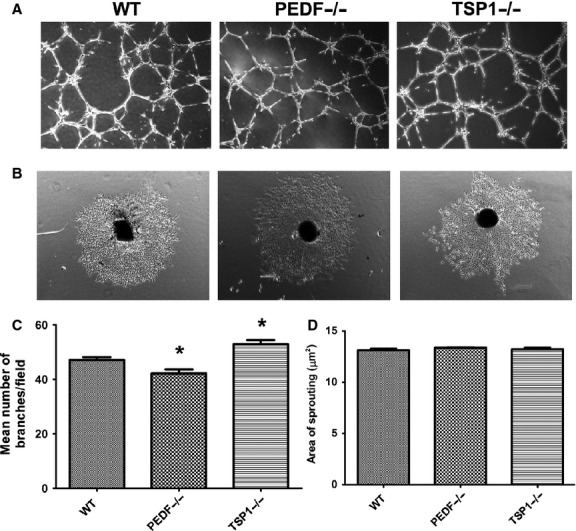
Alterations in capillary morphogenesis of choroidal EC‐RPE cell co‐cultures and RPE‐choroidal explant sprouting. (A) Wild‐type mouse choroidal EC were cocultured with WT, PEDF−/− and TSP1−/− RPE cell on Matrigel, and capillary morphogenesis was monitored after 14 h. Photographs were taken in digital format. (B) Choroidal ex‐vivo sprouting of P21 of wild type, PEDF−/− and TSP1−/− mice. Choroidal explants were prepared and cultured as described in Material and Methods. Images shown here represent results obtained from three different animals with desired genotype (×40). (C) Quantification of the mean number of branch points from six high‐power fields (×40). Please note a decrease in capillary morphogenesis of choroidal EC cultured with PEDF−/− cells compared with wild type cells. In contrast, choroidal EC cocultured with TSP1−/− RPE cells demonstrated enhanced capillary morphogenesis compared to WT RPE cells (*P < 0.05; *n* = 3). (D) Quantification of the mean area of sprouting from eight high‐power fields (×40) from each animal.

Most vascular diseases and their subsequent pathologies are associated with alteration in the cellular oxidative state. Oxidative stress acts as key initiator of inflammation, and is considered a major contributor to the pathogenesis of AMD (Zhou T et al. 2010). Decreased ability of RPE cells to cope with oxidative stress and absence of oxidative defense can promote ocular neovascularization and retinal degeneration (Dong et al. 2009). Using dihydroethidium staining, we examined ROS production at basal level and following incubation with 50 *μ*mol/L H_2_O_2_. PEDF−/− and TSP1−/− RPE cells demonstrated more fluorescent intensity compared with wild‐type cells indicating increased ROS production under basal level or stress condition (Fig. 12A and B). The quantitative assessments of the data are shown in Fig. 12C and D. To further confirm our results, we investigated whether PEDF−/− and TSP1−/− RPE cells exhibit an increase in intracellular accumulation of ROS in vivo. Using wholemount RPE/choroid complexes prepared from wild‐type, TSP1−/−, and PEDF−/− mice the level of oxidative state was determined by 4‐hydroxynonenal (4‐HNE) staining as described in Material and Methods. The 4‐HNE is a key reactive product of lipid peroxidation, and indicator of oxidative stress (Yang et al. 2003). The staining of RPE/choroid complex from PEDF−/− and TSP1−/− mice exhibited increased 4‐HNE staining compared with wild type mice, indicating increased oxidative stress in the absence of PEDF and TSP1 (Fig. 12E). Our results demonstrated that PEDF and TSP1 deficiency is associated with increased oxidative stress in RPE cells. These results are consistent with decreased levels of these proteins in eye samples from AMD patients.

### PEDF and TSP1 deficiency resulted in production of inflammatory mediators in RPE cells

**Figure 13.**
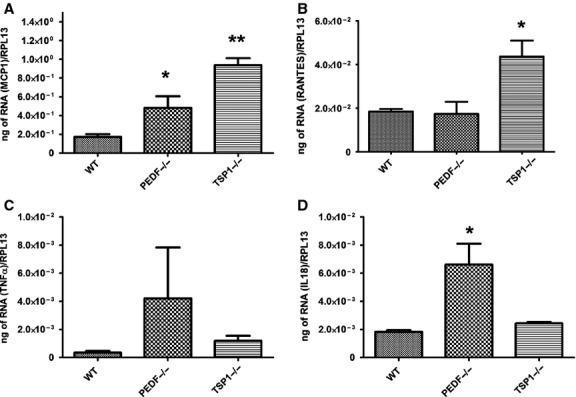
Increased expression of inflammatory mediators in PEDF−/− and TSP1−/− RPE cells. The expression of various inflammatory mediators (MCP‐1, RANTES, TNF‐a, IL‐18) was assessed by quantitative real‐time PCR using RNA from RPE cells as described in the Material and Methods. Please note increased expression of inflammatory cytokines in PEDF−/− and TSP1−/− REP cells compared with wild‐type cells (*P < 0.05, **P < 0.01, *n* = 3). These experiments were repeated with three different isolations of RPE cells with similar results.

Increased oxidative stress in PEDF−/− and TSP1−/− RPE cells suggested potential alteration in the inflammatory response of these cells. We next examined the expression of inflammatory mediators in PEDF−/− and TSP1−/− RPE cells. Figure 13 shows a dramatic increase in expression of monocyte chemotactic protein‐1 (MCP‐1) in both TSP1−/− and PEDF−/− RPE cells compared to the wild‐type cells. PEDF−/− RPE cells exhibited a dramatic increase in production of IL‐18 compared with TSP1−/− RPE and wild‐type cells. Similar trend was observed for TNF‐*α* in PEDF−/− RPE cells, however it was not significant. We also observed increased RANTES production in TSP1−/− RPE cells compared to the PEDF−/− RPE and wild‐type cells. These results indicated that the absence of PEDF and TSP1 is associated with a distinct inflammatory phenotype of RPE cells. Our results are consistent with previous studies establishing that the inflammatory responses in the retina and RPE play a crucial role in the development and progression of AMD and is further supported by detection of decreased levels of TSP1 and PEDF in patients with exudative AMD.

**Figure 14.**
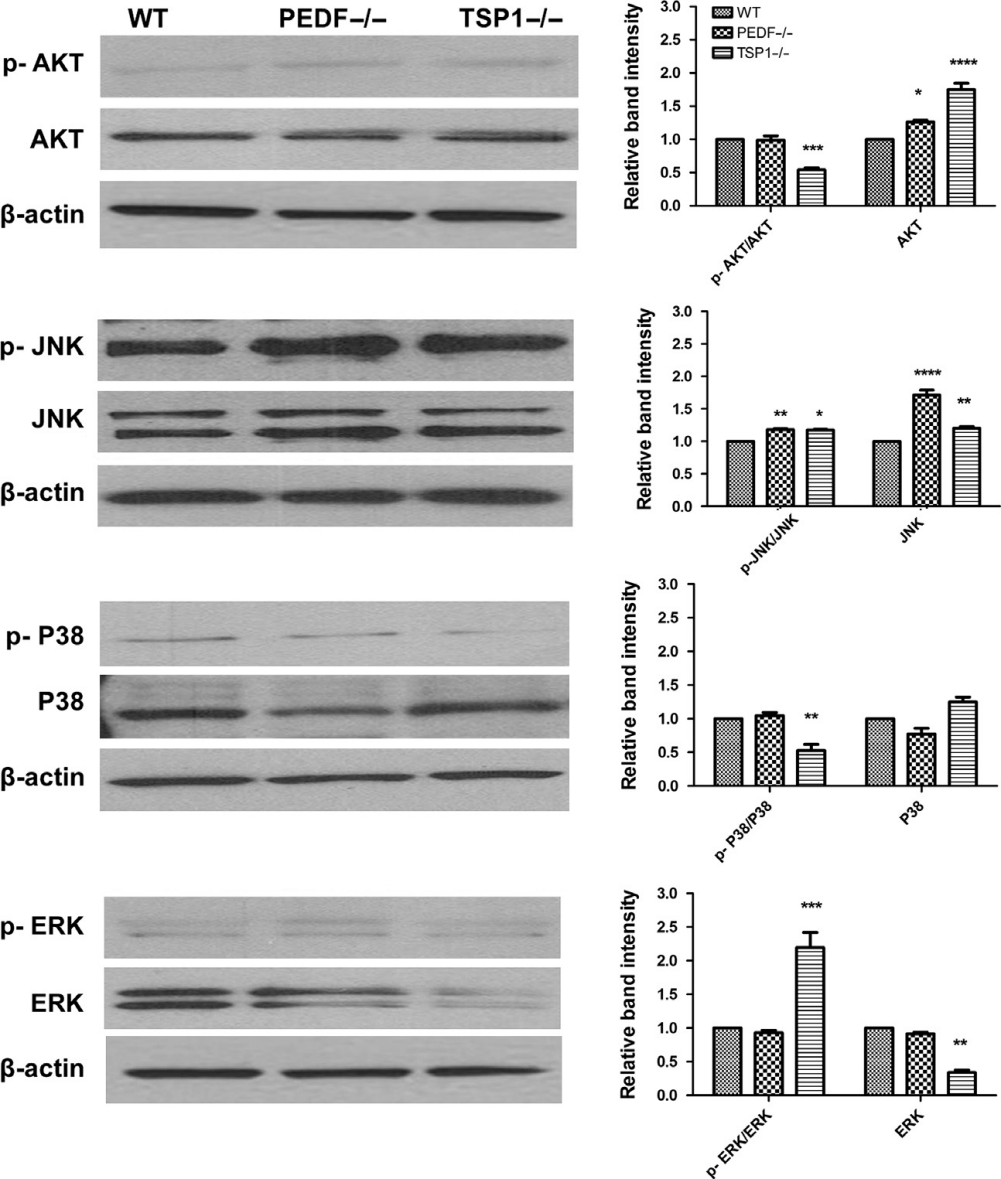
Alterations in the downstream AKT and MAPK signaling pathways. The activation of MAPK and AKT pathways were evaluated by Western blot analysis using specific antibodies as detailed in Material and Methods. A significant change in these pathways were detected in PEDF−/− and TSP1−/− RPE cells compared with WT cells (*P < 0.05, **P < 0.01, ***P < 0.001, ****P < 0.0001; *n* = 3). Please note decreased levels of p‐Akt and p‐p38 in TSP1−/− RPE cells compared to WT and PEDF−/− cells. However, p‐JNK was increased in PEDF−/− and TSP1−/− RPE cells compared with WT cells. TSP1−/− RPE cells also expressed significantly higher levels of p‐ERKs compared to PEDF−/− or WT cells. These experiments were repeated with two different isolations of RPE cells with similar results.

The role of MAPK pathways in oxidative stress signaling is controversial, with MAP kinases JNK and p38 shown to be protective, proapoptotic or not involved (Klettner 2012). The ERK1/2 is considered to be involved in cell proliferation and differentiation. However, it may also contribute to an apoptotic response (Gilley et al. 2009). Increased ERK1/2 activation of RPE cell has been demonstrated in the AMD patients with GA, and its inhibition shown therapeutic benefit (Dridi et al. 2012). TSP1−/− RPE cells demonstrated increased levels of p‐ERK1/2, and lower levels of total ERK1/2 compared to PEDF−/− and wild‐type cells. Thus, there is a potential mechanism whereby decreased TSP1 expression may result in increased ERK1/2 activation in RPE cell of AMD patients. PEDF−/− and TSP1−/− RPE cells exhibited an increase in p‐JNK level compared with wild‐type cells, without affecting the total level of JNK (Fig. 14). The level of p‐p38 was increased in TSP1−/− RPE cells compared with PEDF−/− and wild‐type cells. We did not observe any significant change in the total level of p38 in all cells. Altered proliferation rate in PEDF−/− and TSP1−/− suggested a potential change in AKT activation and/or expression. AKT plays a key role in cell survival and proliferation (Klettner 2012). Here, we observed decreased AKT activation in TSP1−/− RPE cells compared with PEDF−/− and wild‐type cells. However, increased expression of total AKT was detected in PEDF−/− and TSP1−/− RPE cells (Fig. 14). Thus, absence of PEDF and TSP1 were associated with altered MAPK and AKT signaling pathways, which may contribute to the altered cellular functions observed in these cells.

## Discussion

The clinical and pathological signs of AMD, especially the dry form or the geographic atrophy, are associated with degeneration and loss of RPE cells. Although an important role for RPE cell dysfunction has also been demonstrated in exudative form of AMD, these roles may be somewhat distinct and need further elucidation. RPE is a monolayer of specialized epithelial cells which perform a wide variety of functions to preserve retina. However, the detailed mechanisms regulating RPE cell function remain poorly understood. One major function of RPE cells is the maintenance of ocular vascular homeostasis. This is accomplished through a balanced production of pro‐ and antiangiogenic factors including VEGF, PEDF, and TSP1. However, how these factors impact RPE cell function are poorly understood. Here we determined the impact of PEDF and TSP1 deficiency on RPE cell function. Using RPE cells prepared from PEDF−/− and TSP1−/− mice, we showed that the PEDF or TSP1 deficiency resulted in increased proliferation of RPE cells. These cells were also more adherent and less migratory, exhibited increased oxidative stress with minimal impact on basal levels of apoptosis. The PEDF and TSP1 deficiency were also associated with significant changes in production of ECM proteins, cell–cell junctional organization, increased oxidative sensitivity, and altered phagocytic activity. A summary of changes observed in PEDF−/− and TSP1−/− RPE cells compared with wild‐type cells is shown in Table 1. The reexpression of PEDF or TSP1 was sufficient to restore normal migration of PEDF−/− and TSP1−/− RPE cells. Thus, the cell autonomous expression and/or activity of PEDF and TSP1 are essential for appropriate RPE cell function. Furthermore, our results provide a better understanding of the underlying mechanisms, such as altered production of PEDF and TSP1, which contribute to RPE cells dysfunction and pathogenesis of AMD.

**Table 1.** A summary of PEDF and TSP1 deficiency effects on expression of RPE cell markers.

RPE cell marker	WT	PEDF−/−	TSP1−/−	Potential marker function
	Expression	Compared to wild type	Compared to wild type	
RPE 65	+	Similar^1^	Similar^1^	RPE cell specific marker
Bestrophin	+	Similar^1^	Similar^1^	RPE cell specific marker
VACM‐ 1	+	Increased	Decreased	Migration and activation of RPE
VEGF‐R1	+	Increased	Decreased	VEGF receptor, angiogenesis
VEGF‐R2	−	−	−	VEGF receptor, angiogenesis
PDGF‐ R*α*	+	Increased	Highly increased	Cell migration and proliferation
PDGF‐ R*β*	+	Increased	Highly increased	Cell migration and proliferation
Endoglin	−	−	−	Not described
ICAM‐ 1	+	−	Decreased	Inflammation
ICAM‐ 2	+ very low	−	−	Inflammation
CD36	+	Similar^1^	Similar^1^	Phagocytosis
CD47	+	Similar^1^	Decreased	Phagocytosis, TSP1 receptor
MFG‐E8	+	Decreased	Similar ^1^	Phagocytosis, inflammation
PDI	+	Increased	Decreased	Cell viability and protein folding
ZO‐1	+	Highly decreased	Decreased	Cell proliferation, polarity, and paracellular diffusion and permeability
N‐cadherin	+	Similar	Increased	Maintaining tissue integrity and normal morphology, Wnt signaling pathway
*β*‐catenin	+	Highly increased	Increased	Maintaining tissue integrity and normal morphology
P120‐ catenin	+	Increased	Highly increased	Maintaining tissue integrity and normal morphology
FAK	+	Increased	Increased	Focal adhesion, migration
Vinculin	+	Similar	Increased	Actin stress fibers, migration
*α*1	‐	Similar^1^	Similar^1^	Adhesion
*α*2	+	Increased	Decreased	Adhesion
*α*3	+	Decreased	Similar^1^	Adhesion
*α*5	+	Similar^1^	Similar ^1^	Adhesion
*α*V	+	Similar^1^	Similar ^1^	Adhesion
*β*1	+	Similar^1^	Similar^1^	Adhesion
*β*3	+	Similar ^1^	Similar ^1^	Adhesion
*β*5	+	Similar ^1^	Similar^1^	Adhesion
*β*8	+ very low	Similar ^1^	Similar^1^	Adhesion
*α*5*β*1	+	Similar^1^	Similar ^1^	Adhesion
*α*V*β*3	+	Similar^1^	Similar^1^	Adhesion, phagocytosis
Fibronectin	+	Increased (CM, L)	Increased (L), decreased (CM)	Wound closure, inflammatory responses, cell migration, and various developmental processes
Collagen IV	+	Decreased (CM, L)	Decreased (CM, L)	Wound closure, inflammatory responses, cell migration, and various developmental processes
TSP1	+	Decreased (CM, L)	−	Wound closure, inflammatory responses, cell migration, and various developmental processes and angiogenesis
TSP2	−	−	Increased (CM, L)	Similar as TSP1
Tenascin C	+	Similar (L), increased (CM)	Similar (L), increased (CM)	Wound closure, inflammatory responses, cell migration, and various developmental processes
PEDF	+	−	Increased (CM)	Similar as TSP1, antioxidant, Neuro‐protective factor
Periostin	+	Increased (CM, L)	Increased (L), similar (CM)	Wound closure, inflammatory responses, cell migration, and various developmental processes
Opticin	+	Decreased (L), similar (CM)	Increased (L), similar (CM)	Wound closure, inflammatory responses, cell migration, and various developmental processes
Cathepsin‐B	+	Similar	Decreased	Major lysosomal cysteine protease, lysosome activity
Total NOS	+	Similar	Similar	Modulation of angiogenic response
nNOS	−	−	−	Not described
iNOS	+ very low	Increased	−	Modulation of angiogenic response, inflammation
eNOS	+ very low	Decreased	Decreased	Modulation of angiogenic response
VEGF	+	Decreased	Increased	Proangiogenic factor, angiogenesis
p‐AKT/ AKT	+	Similar	Decreased	Cell survival and proliferation
p‐JNK/ JNK	+	Increased	Increased	Protective, proapoptotic or not involved in apoptosis
p‐P38/ P38	+	Similar	Decreased	Protective, proapoptotic or not involved in apoptosis
p‐ERK/ ERK	+	Similar	Increased	Cell proliferation and differentiation

^1^ Not statistically significant.

The ability to gain insight into RPE cell autonomous regulatory mechanisms and function of various genes has been limited by the unavailability of methods for routine culturing of these cells from wild‐type and transgenic mice retina. Here, we established a method to isolate and culture RPE cells from PEDF−/−, TSP1−/− and wild‐type mice crossed with the immorto mouse. These cells allowed us, for the first time, to gain novel insight into the autonomous role PEDF and TSP1 play in regulation of RPE cell function. These cells expressed the RPE cell markers including RPE65 and bestrophin. However, the morphology of PEDF−/− RPE cells was different from wild‐type and TSP1−/− RPE cells, suggesting altered cell–cell and cell–matrix interactions. The morphology of PEDF−/− RPE cells observed here were consistent with those reported in RPE cells cultured in the absence of retinoic acid. The incubation of RPE cells with retinoic acid resulted in a more hexagonal and epitheloid morphology, instead of an elongated and spindle‐like fibroblast appearance. This was mainly attributed to increased levels of PEDF in RPE cells incubated with retinoic acid (Uchida et al. 2005). In addition, the increased level of PEDF in TSP1−/− RPE cells may be responsible, at least in part, for their similar morphology to wild‐type cells.

Although the junctional localization of cell adhesion proteins was not dramatically affected in PEDF−/− and TSP1−/− RPE cells, a significant decrease in the expression of ZO‐1 was observed. The tight junctional protein ZO‐1 is critical for normal cell proliferation, differentiation, and homeostasis of RPE cells. Consistent with our results a marked disruption of the tight‐junction‐associated protein ZO‐1 and abnormal RPE morphology has been demonstrated in human AMD patients. Furthermore, the total level of *β*‐catenin was also increased in PEDF−/− RPE cells but not TSP1−/− RPE cells, which have significant amounts of PEDF. This is consistent with the proposed role of PEDF in inhibition of Wnt/*β*‐catenin signaling pathway (Park et al. 2011). The pathological role of Wnt signaling pathway in AMD has been previously reported (Zhou T et al. 2010). Thus, lack of PEDF may result in increased levels of *β*‐catenin and sustained activation of Wnt signaling pathway and development of AMD. This may be compensated to some extent by downregulation of TSP1 expression and increased PEDF expression, as observed in TSP1−/− RPE cells. However, the detailed regulatory mechanisms involved in coordinated regulation of PEDF and TSP1 need further investigation.

The N‐cadherin level was increased in TSP1−/− RPE cells. However, a significant increase in p120 catenin was observed in both PEDF−/− and TSP1−/− cells. Interestingly, the localization of N‐cadherin to sites of cell–cell contact was decreased in PEDF−/− RPE cells consistent with their elongated spindly morphology and sustained activation of Wnt signaling. The P120 catenin plays an important role in formation of adherence junctions, and its increased production may be a compensatory feedback for defects in cellular adherens and gap junctions. Thus, PEDF and TSP1 may have significant impact on ZO‐1 expression with potential impact on its junctional localization, stability, and barrier function.

The proliferation of RPE cells plays a major role in the development of PVR (Pastor et al. 2002; Tamiya et al. 2010). Previous studies demonstrated that cell junctions and junctional proteins have a significant role in the proliferation of RPE cells. Loss of cell–cell contact contributes to RPE cell proliferation. In addition, downregulation of ZO‐1 is involved in induction of RPE cell proliferation (Georgiadis et al. 2010; Tamiya et al. 2010). Here, we observed a significant increase in proliferation of PEDF−/− and TSP1−/− RPE cells. These results are consistent with lack of appropriate N‐cadherin junctional localization and decreased ZO‐1 expression in PEDF−/− and TSP1−/− RPE cells. Furthermore, our results are supported by studies demonstrating that increased PEDF and TSP1 expression in RPE cells incubated with retinoic acid attenuates their proliferation (Ma et al. 2012; Du et al. 2013).

PEDF was initially identified as a contact inhibitor factor, since its expression was induced when cells reached confluence and stopped growing. PEDF as a trophic factor may also contribute to attenuation of proliferation by decreasing the number of cells entering S phase of the cell cycle and increasing the number of cells entering G0 (Barnstable and Tombran‐Tink 2004). Thus, PEDF may play a significant role in RPE cells proliferation. The impact of TSP1 on cell proliferation is cell specific which is derived from conformational flexibility of TSP1 or different TSP1 receptors (Majack et al. 1986; Adams and Lawler 2011). TSP1 acts as a negative regulator of proliferation in endothelial cells and induces their apoptosis (Tolsma et al. 1993). However, TSP1 expression is essential for PDGF‐mediated proliferation of perivascular supporting cells (Scheef et al. 2009). Here, we observed increased proliferation in TSP1−/− RPE cells, which was concomitant with increased levels of PDGF‐R*β*. Thus, TSP1 expression may also negatively contribute to RPE cell proliferation as occurs in endothelial cell, involving CD36 and CD47 receptors, both of which were expressed in RPE cells. Collectively, our results suggest PEDF and TSP1 expression are involved in modulating RPE cell–cell adhesion and proliferation, and their absence resulted in altered adhesion and increased proliferation.

Alteration in RPE cell migration contributes to some pathological conditions including AMD, proliferative diabetic retinopathy (PDR), and PVR (Dong et al. 2009; Chan et al. 2010). Here, we demonstrated that PEDF and TSP1 deficiency resulted in more adhesive and less migratory phenotype of RPE cells. Furthermore, reexpression of PEDF and TSP1 in PEDF−/− and TSP1−/− RPE cells was sufficient to restore RPE cell migration. These results are contradictory to those reported by Ma et al. (Ma et al. 2012) who showed increased PEDF expression as a result of over expression of microphthalmia‐associated transcription factor or exogenous PEDF inhibited RPE cell migration. These contradictory results may be attributed to the different levels of PEDF in these studies and require further evaluation.

Cell migration is influenced by cell–ECM protein interactions. Thus, the abrogated migratory features of PEDF−/− and TSP1−/− RPE cells are consistent with their enhanced adhesion. Although, lack of PEDF and TSP1 was associated with minimal changes in expression of integrins, the possibility of alteration in avidity and affinity of integrins, however, cannot be excluded and requires further investigation. We detected altered expression of ECM proteins in PEDF−/− and TSP1−/− RPE cells, which may contribute to the altered migratory and adhesive features of RPE cells with PEDF and TSP1 deficiency. Taken together, these observations suggest that PEDF and TSP1 play key roles in cell–cell and cell–ECM interactions, which modulate proproliferative and promigratory signaling pathways of RPE cells. Additionally, altered ECM composition of RPE cells due to PEDF and TSP1 deficiency may result in defective ocular angiogenesis such as abnormal CNV in AMD.

VEGF is a proangiogenic factor with prosurvival activity and maintenance of RPE integrity (Ford et al. 2011). The increased production of VEGF has been identified as essential in the development and progression of AMD and CNV. Here, we demonstrated that TSP1−/− cells expressed significantly higher levels of VEGF while, PEDF−/− cells produced significantly lower amounts of VEGF compared to wild‐type cells. The increased level of VEGF in TSP1−/− RPE cells is consistent with the proangiogenic state of the retinal vasculature in global TSP1 null retina (Hiscott et al. 1996a,b) and enhanced neovascularization observed in mouse laser‐induced CNV model (Wang et al. 2012).This was mainly attributed to increased recruitment of microglial cells to the site of lesions, perhaps through enhanced inflammatory signaling through the CCR5/RANTES (CCL5) axis in the absence of TSP1 (Zhou T et al. 2010; Lavalette et al. 2011). This notion is supported by observed increased production of RANTES/CCL5 in TSP1−/− RPE cells compared to PEDF−/− or wild‐type mice. Furthermore, we have observed no significant differences in degree of CNV in PEDF−/− mice subjected to laser‐induced CNV (our unpublished results). The reason for significant decrease in VEGF level in PEDF −/− RPE cells is not clear, and is in contrast to reports that increased VEGF level is associated with increased oxidative stress and decreased level of PEDF in diabetic ocular samples (Boehm et al. 2003). These results are, however, consistent with our previous findings where no association was observed between diabetic retinopathy where VEGF level is shown to be increased without a major effect on PEDF levels (Wang et al. 2009). Altogether our results demonstrate that PEDF and TSP1 may share some common pathways in regulation of RPE cell function, they may behave differently development and progression of AMD, perhaps as a result of their different impact on VEGF production in RPE cell.

In summary, consistent with previous studies (Adams and Lawler 2004; Barnstable and Tombran‐Tink 2004), we demonstrated that PEDF and TSP1 play key roles in RPE cell function and subsequently in pathogenesis of AMD. We showed TSP1 and PEDF deficiency in RPE cells resulted in their enhanced proliferation and reduced migration. These changes were associated with alterations in adhesion and production of various ECM proteins. Significant changes in the expression and localization of junctional proteins were also observed. The alterations in MAPK and AKT signaling pathways were consistent with abnormalities observed in adhesion, oxidative stress, and sensitivity to oxidative challenge. Collectively, our results imply that PEDF and TSP1 play a critical role in regulation of the RPE cell proliferation and migration through alterations of their inflammatory and oxidative state.

## Conflict of Interest

No conflict of interest, financial or otherwise, is declared by the authors.
